# The Metagenome-Derived Enzymes LipS and LipT Increase the Diversity of Known Lipases

**DOI:** 10.1371/journal.pone.0047665

**Published:** 2012-10-24

**Authors:** Jennifer Chow, Filip Kovacic, Yuliya Dall Antonia, Ulrich Krauss, Francesco Fersini, Christel Schmeisser, Benjamin Lauinger, Patrick Bongen, Joerg Pietruszka, Marlen Schmidt, Ina Menyes, Uwe T. Bornscheuer, Marrit Eckstein, Oliver Thum, Andreas Liese, Jochen Mueller-Dieckmann, Karl-Erich Jaeger, Wolfgang R. Streit

**Affiliations:** 1 Department of Microbiology and Biotechnology, Biocenter Klein Flottbek, University of Hamburg, Hamburg, Germany; 2 Institute of Molecular Enzyme Technology, Heinrich Heine University Duesseldorf, Research Center Juelich, Juelich, Germany; 3 European Molecular Biology Laboratory (EMBL) Hamburg Outstation, c/o Deutsches Elektronen-Synchrotron (DESY), Hamburg, Germany; 4 Institute of Bioorganic Chemistry, Heinrich Heine University Duesseldorf, Research Center Juelich, Juelich, Germany; 5 Bioprocess Development Consumer Specialties and Biocatalysis Biotechnology, Evonik Industries AG, Essen, Germany; 6 Department of Biotechnology & Enzyme Catalysis, Institute of Biochemistry, Greifswald University, Greifswald, Germany; 7 Institute of Technical Biocatalysis, Hamburg University of Technology, Hamburg, Germany; Auburn University, United States of America

## Abstract

Triacylglycerol lipases (EC 3.1.1.3) catalyze both hydrolysis and synthesis reactions with a broad spectrum of substrates rendering them especially suitable for many biotechnological applications. Most lipases used today originate from mesophilic organisms and are susceptible to thermal denaturation whereas only few possess high thermotolerance. Here, we report on the identification and characterization of two novel thermostable bacterial lipases identified by functional metagenomic screenings. Metagenomic libraries were constructed from enrichment cultures maintained at 65 to 75°C and screened resulting in the identification of initially 10 clones with lipolytic activities. Subsequently, two ORFs were identified encoding lipases, LipS and LipT. Comparative sequence analyses suggested that both enzymes are members of novel lipase families. LipS is a 30.2 kDa protein and revealed a half-life of 48 h at 70°C. The *lipT* gene encoded for a multimeric enzyme with a half-life of 3 h at 70°C. LipS had an optimum temperature at 70°C and LipT at 75°C. Both enzymes catalyzed hydrolysis of long-chain (C_12_ and C_14_) fatty acid esters and additionally hydrolyzed a number of industry-relevant substrates. LipS was highly specific for (*R*)-ibuprofen-phenyl ester with an enantiomeric excess (*ee*) of 99%. Furthermore, LipS was able to synthesize 1-propyl laurate and 1-tetradecyl myristate at 70°C with rates similar to those of the lipase CalB from *Candida antarctica*. LipS represents the first example of a thermostable metagenome-derived lipase with significant synthesis activities. Its X-ray structure was solved with a resolution of 1.99 Å revealing an unusually compact lid structure.

## Introduction

Lipolytic enzymes including lipases (EC 3.1.1.3) and carboxylesterases (EC 3.1.1.1) are important biocatalysts employed for a large number of biotechnological applications [1]–[3]. Many lipases exhibit high chemo-, regio- and enantioselectivity and are tolerant against organic solvents which makes them even more attractive for organic synthesis reactions [4], [5].

A variety of biotechnologically interesting reactions require elevated temperatures and thermostable rather than mesophilc enzymes [6]–[8]. While the general prediction of thermostabilty of an enzyme entirely based on the deduced amino acid sequence of a protein is perhaps not reliable [9], several traits appear to be associated with thermostable proteins. Mainly disulfide bonds and intrahelical salt bridges are more frequently observed in thermostable enzymes. Furthermore, the overall composition of amino acids appears to be of importance for the thermostability and especially polar residues that form additional hydrogen bonds appear to be of importance. Further the use of charged residues to form additional ionic interactions is yet another key trait of thermostable enzymes [10], [11]. Recently it was also suggested that the frequency of Asn-Glu could be a factor to distinguish between mesophilic and thermophilic proteins [12]. Esterification at higher temperatures offers the advantages that the reactions can take place at higher rates and the use of organic solvents can be avoided. The respective biocatalysts thus need to be thermotolerant showing high activity at elevated temperatures above 70°C. Ideal resources for such enzymes are microbes living under extreme conditions [13], [14]. While in the last decade many thermostable enzymes - including a significant number of esterases - have been uncovered, the number of truly thermophilic and bacterial lipases is still limited with less than ten thermostable bacterial lipases being characterized to date. Among them is a remarkably stable enzyme from *Thermoanaerobacter thermohydrosulfuricus* (LipTth) and a lipase from *Caldanaerobacter subterraneus* (LipCst) [15]. Further, several thermoactive lipases have been reported in the genus *Geobacillus* [16]–[18]. Although these enzymes are active at high temperatures, they appear to be less stable over time when incubated at elevated temperatures. Furthermore, thermostable lipases have been reported in different *Thermus* isolates [19], [20] and were recently expressed in thermophilic yeasts [21], [22]. A thermostable esterase from *Thermus scotoductus* has been reported that was partially biochemically characterized [23]. Finally, two thermostable lipases have been reported from *Thermosyntropha lipolytica*, an anaerobic, thermophilic, alkali-tolerant bacterium that grows syntrophically with methanogens on lipids [24]. Both enzymes from this microbe were active at temperatures of >90°C and showed remarkable half-life times at 100°C.

Furthermore, a number of moderately thermostable lipases that originated from fungi have been described and some of them have been analyzed or optimized through evolutive strategies [25]–[28]. Of those, the most frequently used and best characterized moderately thermostable lipase is CalB, which originates from the mesophilic yeast *Candida antarctica* [25]. By applying several rounds of protein engineering methods, the thermal stability of CalB was improved greatly [29]–[31]. While these enzymes were all derived from cultivable bacteria or fungi, surprisingly, no truly thermostable lipases acting on long-chain *p*NP-esters with temperature optima of 70°C or higher have been reported using a metagenome-based approach since the first discovery of lipolytic enzymes from metagenomes over ten years ago [32], [33].

Metagenome-based technologies for the identification of novel biocatalysts have been applied very successfully within the last decade and have resulted in the identification of numerous novel biocatalysts [34], [35]. However, the basic steps of accessing non-cultivated microorganisms have been outlined earlier and include the isolation of environmental DNAs, cloning into small or large insert vectors and amplification of these libraries in a suitable host [36], [37]. The libraries are then screened using a wide array of different methods. With respect to the screening and detection of lipases and esterases in metagenomes [38], more than 100 metagenomic enzymes have been reported and in part characterized [33]. Some of these enzymes reveal remarkable traits that are potentially useful for biotechnological applications and have broadened our knowledge on the diversity of lipases. Perhaps the first true lipase reported from a metagenome source was described by Henne and colleagues [32]. Unfortunately, only a fraction of the metagenome-derived lipolytic enzymes has been characterized in more detail concerning their structural features [39]–[43].

Here, we have used metagenome-based technologies to identify and characterize novel bacterial lipolytic enzymes which catalyze both hydrolysis and esterification reactions at temperatures above 70°C. Metagenomic libraries from enriched soil and water samples were constructed and screening revealed two novel lipases designated LipS and LipT which showed a high temperature optimum and also a high stability against thermal denaturation. These lipases were biochemically characterized and the X-ray structure of LipS was solved at a resolution of 1.99 Å in its apo form and together with spermidine. Thus, LipS belongs to the first metagenomic lipases that have been analyzed by crystallographic methods so far.

## Materials and Methods

### Environmental Samples and Enrichment Cultures

Ten soil and water samples were collected from different sites at the Botanical Garden (Klein Flottbek, Hamburg, Germany, 53°33′44.56′ ´N, 9°51′40.11′ ´E). The sample sites included topsoil that consisted mainly of sand as well as humus-rich soil. Water samples were taken from sweet water brooks and ponds. Approximately 0.5 g of each soil sample and 0.5 ml of each liquid sample were then mixed in a 100 ml Erlenmeyer flask containing 50 ml of mineral salt medium (MSM) and incubated overnight at room-temperature and 150 rpm in order to detach bacterial cells from soil and plant particles. After sedimentation of these particles by gravity, the samples were used to inoculate mineral salt medium (MSM) in a 2 l glass bottle composed of 0.8 l H_2_O, 0.1 l solution 1 and 0.1 l solution 2 [solution 1 (1 l, 10×): 70 g Na_2_HPO_4_×2 H_2_O, 20 g KH_2_PO_4_. Solution 2 (1 l, 10×): 10 g (NH_4_)_2_SO_4_, 2 g MgCl_2_×6 H_2_O, 1 g Ca(NO_3_)_2_×4 H_2_O]. The medium was supplemented with pyruvate (0.1% w/v), olive oil (1% v/v), vitamins [100 ml, 1000×: 1 mg biotin, 10 mg nicotinic acid, 10 mg thiamin-HCl (vitamin B1), 1 mg *p*-aminobenzoic acid, 10 mg Ca-D-(+)-pantothenic acid, 10 mg vitamin B6 hydrochloride, 10 mg vitamin B12, 10 mg riboflavin, 1 mg folic acid] and trace elements [44]. The enrichment culture was maintained at 65°C and aerated with 120 rpm on a magnetic stirrer.

For the second enrichment, water samples were taken from a heating system in the Biocenter Klein Flottbek (Hamburg, Germany). The temperature of the water at the time of sampling was above 50°C. The medium [modified medium D [45]] contained tryptone and yeast extract (0.1% w/v each) as well as trace elements [46]. This *Thermus*-enrichment culture was inoculated with 20% of water sample (v/v) and incubated at 75°C in a 2 l glass bottle on a magnetic stirrer with 200 rpm for several weeks. Both enrichment media were refilled with autoclaved H_2_O_dest_ on a regular basis to maintain the initial volume.

No specific permits were required for the described field studies as the Botanical Garden and the Biocenter Klein Flottbek are non-protected areas concerning soil and water samples and owned by the University of Hamburg. The samples did not involve endangered or protected species.

### E. coli Culture Conditions

*E. coli* strains were grown aerobically at 37°C on Luria-Bertani (LB) medium supplemented with appropriate antibiotics [47]. *E. coli* clones and constructs are listed in TABLE S1.

### DNA Isolation, 16S rRNA Analysis and Library Construction

After three weeks of incubation, cells from the enrichment cultures were harvested by centrifugation. Genomic DNA was isolated by using a phenol/chloroform method with TE-buffer containing sucrose [10 mM Tris-HCl, 1 mM EDTA and 20% sucrose (w/v)], lysozyme solution (1 mg/ml in TE-buffer) and proteinase K solution [1 mg/ml, 20% SDS (w/v), 1 mg/ml RNase].

For phylogenetic characterization of the enrichments, bacterial 16S rRNA genes were amplified using the standard primers 616V (5′-AGAGTTTGATYMTGGCTCAG-3′) and 1492R (´5-CGGYTACCTTGTTACGAC-3′). The amplified genes were ligated into pDrive cloning vector and transformed in competent *E. coli* DH5α cells by heat shock. 16S rDNA was sequenced with automated sequencing ABI377 technology following the manufacturer’s instructions.

Libraries were constructed with the cosmid vector pSuperCos which carries ampicillin and neomycin resistance genes and phage packaging mixes which were both supplied within the Gigapack® III Gold Packaging Extract kit (Stratagene, La Jolla, CA, USA). Construction was carried out according to the manufactureŕs instructions. Genomic DNA fragments with a size of 20–40 kb obtained after partial *Bsp*143I digestion were ligated into the *Bam*HI restriction site of the cosmid vector before phage-infection of *E. coli* Epi100 cells was performed. Cosmid clones were grown on LB agar supplemented with 100 µg/ml ampicillin.

### Screening of Lipolytic Clones

*E. coli* clones were tested for lipolytic activity by transferring them on LB agar plates containing tributyrin (TBT, 1% vol/vol) as indicator substrate [48]. In order to detect active clones, the cosmid clones were grown at 37°C overnight; then a further incubation for 1–3 days at 56°C followed. The second incubation step was introduced to slowly lyse the *E. coli* cells and to release those enzymes that are active on TBT at elevated temperatures and produce a clear halo. In a microtiter plate scale, clones were grown in a 96 deep-well plate containing 1.2 ml of LB with ampicillin. After incubation for 16 to 24 h at 37°C and 250 rpm, cells were harvested by centrifugation and the supernatant was discarded. Cells were lysed by 1 h incubation with 125 µl/well 0.1 M potassium phosphate buffer (PB) pH 8.0 containing lysozyme (10 mg/ml) at 37°C. Cell debris was collected by centrifugation for 10 min at 3,600 rpm. In a 96 well microtiter plate, 10 µl of the crude cell extract were incubated with 190 µl of PB (0.1 M, pH 8.0) that contained either 1 mM 4-nitrophenyl (*p*NP) butyrate or dodecanoate. The samples were incubated for 30 min at 56°C and subsequently, the extinction of 4-nitrophenol released from the substrate was measured spectrophotometrically in a microtiter plate reader (Benchmark, Bio-Rad, Hercules, CA, USA) at 405 nm against an enzyme free blank.

Cosmid DNA was isolated from the positive clones obtained in the initial screening, retransformed in *E. coli* DH5α and the resulting clones examined with the same type of assay for esterase/lipase activity in order to avoid false positive clones.

### Subcloning and in vitro Transposon Mutagenesis

For the identification of ORFs encoding lipolytic activity, the positive cosmid clones were subcloned with *Eco*RI, *Hind*III or *Sac*I, ligated into pTZ19R, which carries a chloramphenicol resistance gene, and transformed into *E. coli* DH5α. The subclones were streaked onto LB agar plates with TBT and screened for hydrolytic activity. On positive subclones, *in vitro* transposon mutagenesis using the EZ::TN™ <KAN-2> transposon kit (Epicentre, Madison, Wisconsin, USA) was carried out following the manufacturer’s instructions. Clones harboring a transposon in the responsible gene were screened negative on TBT containing agar plates. With the inserted priming sites of the transposon, the corresponding gene was sequenced by automated sequencing ABI377 technology following the manufacturer’s instructions. Alternatively, the inserts of the subclones were sequenced with the vector specific primers M13 for (´5-GTAAAACGACGGCCAGT-3′) and M13 rev (5′-CAGGAAACAGCTATGACC-3′).

### Cloning and Expression of lipS and lipT

Gene sequences were amplified from cosmid DNAs by PCR in 35 cycles with the primer pairs pCos9D12_for (5′-CATATGAGCCGGAAAAGCAGG-3′) and pCos9D12_rev (5′-AAGCTTGCTGTGCTTCCGGATGAAC-3′) for the amplification of *lipS* and pCos6B1_for (5′-CATATGCGGCGGTTACTAGCCTTGC-3′) and pCos6B1_rev (5′-AAGCTTCCGCACCCTAGGCGCCGCC TTC-3′) for *lipT*. Primers were designed to introduce a 5′-*Nde*I and a 3′-*Hind*III restriction site into the cloned fragments. The PCR fragments were ligated into pDrive cloning vector (Qiagen, Hilden, Germany), cut with *Nde*I and *Hind*III and ligated into pET21a (Novagen, Merck, Darmstadt, Germany), which has an ampicillin resistance gene and a His-tag coding sequence for the C-terminus of the corresponding protein. Plasmids containing *lipS* and *lipT* gene sequences were designated *lipS*::pET21a and *lipT*::pET21a, respectively. To confirm that the correct genes had been amplified from the original cosmid DNA, the PCR fragments cloned into pET21a were sequenced. Competent *E. coli* BL21 (DE3) cells were transformed by heat shock with *lipS*::pET21a and *lipT*::pET21a for the overproduction of the corresponding proteins. Cultures were grown at 17°C and 250 rpm for 6–8 h until an optical density at 600 nm of 0.8 was reached. The production of the recombinant proteins was then induced by the addition of 1 mM isopropyl-β-D-thiogalactopyranoside (IPTG). After 16 h, the cells were harvested by centrifugation and disrupted by French pressure cell and ultrasonication in order to purify LipS and LipT from soluble fractions. Cell extracts were incubated with Ni-NTA Agarose (Qiagen, Hilden, Germany), loaded on columns and affinity chromatography was carried out according to the manufactureŕs protocol. Protein containing elution fractions were then dialyzed overnight against 0.1 M PB (pH 8.0). The proteins were analyzed by SDS polyacrylamide gel electrophoresis using 12 or 15% (w/v) gels and Western-immunoblotting using 6-His-specific antibodies.

### Catalytic Activity Toward 4-nitrophenyl (pNP) Substrates

Enzyme activity studies were performed by incubating the enzymes with 1 mM *p*NP-substrate in 0.1 M PB (pH 8.0) at assay temperatures of 70°C (LipS) or 75°C (LipT), unless otherwise indicated. The reaction was measured against an enzyme-free blank to subtract auto-hydrolysis by spectro-photometrical quantification of the released 4-nitrophenol at 405 nm [molar extinction coefficient ε (0.1 M PB pH 8.0) = 19,454 M^−1^ cm^−1^, ε (0.1 M PB pH 7.0) = 10,400 M^−1^ cm^−1^]. One unit is defined as the amount of enzyme that catalyzes the formation of 1 µmol 4-nitrophenol per minute. Enzyme activity was tested against different *p*NP-acyl esters [butyrate (C4), hexanoate, octanoate, decanoate, dodecanoate, myristate (C14), palmitate (C16) and stearate (C18), Sigma]. Above 70°C, even long-chained *p*NP esters (C16–C18) were sufficiently soluble, so that no detergents were added. The temperature optima of LipS and LipT were determined with *p*NP-dodecanoate as substrate at temperatures ranging from 20 to 90°C for 10 min. To study the thermal stability of the enzymes, LipS and LipT were incubated at 70 and 90°C, respectively, for up to 72 hours and their residual activity was measured using *p*NP-dodecanoate (1 mM final concentration) by incubation for 20 min at 70°C for LipS and 75°C for LipT.

The pH optimum of LipS and LipT was investigated with buffers of different pH values, that were adjusted at 70°C [pH 5–5.6, citrate buffer (0.05 M); pH 5.6–8, PB (0.1 M); pH 8–9, Tris-HCl (0.1 M); pH 9–10.6, glycine/NaOH (0.1 M)]. Enzyme activity was measured with *p*NP-decanoate as substrate.

LipS and LipT were tested for their stability and activity in the presence of metal ions, inhibitors, detergents and solvents. After 1 h incubation with these substances at room temperature, residual enzyme activities were determined at 70°C or 75°C and at pH 8.0 in 0.1 M PB by using *p*NP-decanoate or -dodecanoate as substrates.

As metal ions, Ca^2+^, Co^2+^, Cu^2+^, Fe^3+^, Mg^2+^, Mn^2+^, Rb^2+^ and Zn^2+^ were used with a concentration of 1 or 10 mM in 0.1 M PB. EDTA (ethylenediaminetetraacetic acid), DTT (dithiothreitol) and PMSF (phenylmethyl-sulfonyl fluoride) were used as enzyme inhibitors with 1 or 10 mM concentration in PB. In order to examine the stability against detergents, SDS (sodium dodecyl sulfate), Triton X-100 and Tween 80 were applied with 1 or 5% concentration (w/v, v/v) in 0.1 M PB pH 8.0.

The stability of LipS and LipT in various organic solvents was studied using dimethyl sulfoxide (DMSO), isopropanol, methanol, dimethylformamide (DMF), acetone, acetonitrile and ethanol at final concentrations of 10% or 30% (v/v) in 0.1 M PB pH 8.0.

The substrate range of the two enzymes was tested with the following achiral or racemic *p*NP-esters at a final concentration of 0.5 mM in 0.1 M PB pH 8.0∶2-phenylpropanoate, 3-phenylbutanoate, cyclohexanoate, 2-(3-benzoylphenyl) propanoate, 2-naphthoate, 1-naphthoate, adamantanoate and 2-(4-isobutylphenyl)-N-propanamide ester. Activity was measured at 405 nm after 10, 20 and 30 minutes incubation at 70°C.

Activity on chiral *p*NP-esters was analyzed with (*S*)-/(*R*)-2-methyldecanoic acid ester [49], [50], (*S*)-/rac-/(*R*)-2,3-dihydro-1*H*-indene-1-carboxylate [“Indancarboxylic acid ester”, [51]], (*S*)-/rac-ibuprofen-ester and (*S*)-/rac-/(*R*)-naproxen-ester [52], [53].

Enzyme activity on these *p*NP-esters with 0.33 mM final concentration was measured at 410 nm after incubation for up to 40 min at 60 and 65°C in 0.05 M Soerensen buffer pH 8.0 containing 0.1% (w/v) gum arabic, 5 mM sodium deoxycholate and 10% DMSO. Controls concerning these additives did not reveal a significant effect on enzyme activity.

### HPLC-MS Analysis of LipS on pNP and Phenyl Esters of Ibuprofen

To determine enantioselectivity referring to the *p*NP and the phenyl ester of ibuprofen, kinetic resolution has been carried out in analytical scale: 17.32 ml potassium buffer (0.1 mM, pH 8.0) were mixed with 2 ml DMSO and 0.66 ml of a substrate stock solution (10 mM in DMSO). 652 µg LipS were added and the reaction was shaken at 60°C for 30 min. The reaction was stopped by adding 8 ml 2 M HCl and followed by immediate extraction with methyl *tert*-butyl ether (MTBE, 2×20 ml). The solvent was removed under reduced pressure. The extracted ibuprofen was converted to the corresponding methyl ester by adding a 0.5 M diazomethane solution in diethyl ether. The solvent was removed under reduced pressure. The *ee* was determined by HPLC (Dionex) using a chiral stationary phase: Chiralpak IA (Daicel), 99.8∶0.2 (*n*-heptane:isopropanol), 0.5 ml/min, 225 nm, *t_R_*(*S*) = 10.23 min, *t_R_*(*R*) = 11.17 min. The *ee* of the phenyl ester was determined using the same conditions as the methyl ester [*t_R_*(*S*) = 16.32 min, *t_R_*(*R*) = 18.07 min]. Because the enantiomers of the *p*NP ester could not be separated by chiral HPLC, the *ee* was determined by measuring the g factor (dissymmetry factor) with achiral HPLC with CD detector [54]–[56]. Column: Hyperclone ODS C18, conditions: 90∶10, CH_3_CN:H_2_O, 0.5 ml/min, 220 nm, *t_R_*(ibuprofen) = 2.7 min, *t_R_*(*p*NP ester) = 4.5 min. Calculation of the enantioselectivity (*E*) value was performed by the method of *Faber et al*. [57].

### Catalytic Activity Measured using Titration Assays

Tributyrin, triolein and polyglycerol-3-laurate were chosen as substrates for LipS and LipT to study activity on triglycerides using an automated titrator (Titrando 842 with Dosino 800, Metrohm, Filderstadt, Germany) and the pH-stat method. The substrate concentrations of the triglycerides ranged from 5 to 50 mM and of polyglycerol-3-laurate from 0.5 to 7.5% (w/v) in 2 mM Tris-HCl buffer pH 7.0. The substrate was emulsified with an automated stirrer (stirrer 802, Metrohm, Filderstadt, Germany) in the reaction vessel. The reaction was performed at 60°C, below the optimal temperature of the enzymes, in order to avoid autohydrolysis of the substrates. In order to have a control rate and for determination of autohydrolsis, the pH of the substrate solution was measured at 60°C for 5 min before the enzyme was added. The consumption rate of 20 mM KOH which was used to keep the pH at 7.0 indicated enzyme activity and was used to calculate the specific activity expressed in units per milligram of enzyme (U/mg). One unit was the amount that produced 1 µmol of fatty acid per minute under the specified assay conditions.

### Esterification (Propyl Laurate) Assay

The propyl laurate assay was applied with 1-propanol and lauric acid as well as 1-tetradecanol and myristic acid as substrates for LipS. Both reactants were incubated in equimolar conditions (20 mmol) at 70°C together with 15 mg of lyophilized enzyme in a closed bottle under slow rotation. After 0, 24 and 48 h, the acid values of the reaction mixtures were determined by titration of a 0.5 g sample solved in 20 ml of toluene against 0.5 M KOH_ethanol_ with phenolphthalein as pH indicator. The resulting acid values were used for the calculation of propyl laurate/tetradecyl myristate units per mg of enzyme. One unit was defined as 1 µmol of propyl laurate or tetradecyl myristate formed per minute by the enzyme under above mentioned assay conditions.

### Enzyme-catalyzed Kinetic Resolution of Four Acetates of Secondary Alcohols

Three racemic acetates, i. e. 1-phenyl-1-propyl acetate, 1-phenyl-2-butyl acetate and 1-phenyl-2-pentyl acetate, were synthesized from the corresponding racemic alcohols as already described [58], [59] except for 1-phenyl-1-ethyl acetate, which was commercially available. For the kinetic resolution, 10 mM acetate were added to a 1 ml solution containing 0.25 mg pure enzyme dissolved in PB (0.1 M, pH 7.0) and were mixed in a thermoshaker (Eppendorf, Hamburg, Germany) with 13,000 rpm at 70°C. Samples (100 µl) were taken at different time intervals and extracted twice with 100 µl dichloromethane. The combined organic layers were dried over anhydrous sodium sulfate and the organic solvent was removed in a nitrogen stream. The enantiomeric excess (%*ee*) of substrate and product were determined by gas chromatography as described earlier [58], [59] [GC, Shimadzu GC-14A gas chromatograph, column: heptakis(2,6-*O*-methyl-3-*O*-pentyl)-β-cyclodextrin (Machery-Nagel, Düren, Germany); carrier gas H_2_; flame ionization detector]. The retention times were as follows: 1-phenyl-1-propyl acetate *t_R_*(*S*) = 5.7 min, *t_R_*(*R*) = 6.9 min; 1-phenyl-1-propanol *t_R_*(*S*) = 11.8 min, *t_R_*(*R*) = 12.7 min; 1-phenyl-1-ethyl acetate *t_R_*(*S*) = 3.9 min, *t_R_*(*R*) = 5.3 min; 1-phenyl-1-ethanol *t_R_*(*S*) = 6.2 min, *t_R_*(*R*) = 6.8 min; 1-phenyl-2-butyl acetate *t_R_*(*S*) = 17.5 min, *t_R_*(*R*) = 19.3 min; 1-phenyl-2-butanol *t_R_*(*S*) = 21.9 min, *t_R_*(*R*) = 23.6 min; 1-phenyl-2-pentyl acetate *t_R_*(*S*) = 31.5 min, *t_R_*(*R*) = 31.9 min; 1-phenyl-2-pentanol *t_R_*(*S*) = 37.5 min, *t_R_*(*R*) = 37.5 min. *E*-value and conversion were calculated from the *ee* of substrate and product according to Chen *et al*. [60].

### Classification of LipS and LipT

Amino acid sequences of the eight major bacterial lipase/esterase families [61] were obtained from the NCBI GenBank database (see supplementary TABLE S2). Independent alignments for all families were constructed using T-coffee [62]. All metagenome derived lipase/esterase sequences were sorted into the eight families based on alignment scores (see supplementary TABLE S3) and visual inspection of the respective alignments. Sequences homologous to LipS and LipT were retrieved from the NCBI GenBank database. The LipS and LipT groups of sequences as well as 11 other metagenome sequences could not unequivocally be assigned to any of the known lipase families. Therefore, all of those sequences were compared to each other and when feasible sorted into a subgroup. In conclusion, those sequences constitute the LipS and LipT family as well as five additional unknown metagenome lipase/esterase sequence families (UF1-5).

Due to low sequence conservation between the different bacterial lipase/esterase sequence families, the independently constructed alignments had to be combined into a final dataset using Genedoc [63]. Tree reconstruction was carried out using the RaxML webserver [http://phylobench.vital-it.ch/raxml-bb/, [64]]. Tree viewing and editing was carried out using ATV [65] or TreeIllustrator v0.52 [66].

### Crystallographic Analyses

LipS was crystallized and crystallographic data sets were collected and reduced as described previously [67]. The structure of wild-type LipS (LipS-WT) was solved by molecular replacement (MR) using the structure of carboxylesterase Est30 from *Geobacillus stearothermophilus* (PDB code 1TQH) as a model. The search was carried out with Molrep [68], which identified 4 molecules per asymmetric unit (a.u.), as expected from a Matthews parameter of 2.6 [69]. Iterative cycles of manual rebuilding in COOT [70] with crystallographic refinement in Refmac5 [71] converged at a final model at 1.99 Å resolution of good quality. The last rounds of refinement were done without non-crystallographic symmetry (NCS) restraints and with individual, isotropic B-factors.

A second construct of LipS with His_6_-tag at the C-terminus (LipS-H6) crystallized in SG P4_2_2_1_2 and diffracted X-ray radiation to 2.80 Å resolution. Those data were phased by MR using the refined structure of LipS-WT solved in SG P4. Crystals in this SG contained only 2 molecules per a.u. Refinement and quality statistics of both models are given in TABLE S4. The PyMOL software was used for structural alignment, analysis, secondary structure assignment and visualization of protein structures [72].

### Data Submission to Public Databases

The DNA sequences of *lipT* and *lipS* were deposited at GenBank under the accession numbers JQ028671 and JQ028672, respectively. The crystallographic data were submitted with the PDB database under the accession codes 4FBL and 4FBM.

## Results

### Enrichment Strategies and Construction of Metagenomic Libraries

From two different habitats, altogether 11 samples were taken and used to inoculate two different enrichment cultures. Bacteria from a water sample of a heating water system were grown at 75°C on medium D, while bacteria from the ten different soil and water samples of the Botanical Garden were enriched at 65°C on MSM supplemented with pyruvate and olive oil. After one and two weeks, respectively, visible turbidity appeared in the culture media. After three weeks of incubation, the cell density was high enough so that cells were harvested and sufficient genomic DNA could be isolated for library construction. The growth of the organisms appeared to be rather slow, probably because of the relatively low cell density of the inoculum that was used. The microbial communities were characterized on a phylogenetic level by amplification and sequencing of 16S rRNA genes. The gene sequences were aligned with nucleotide sequences deposited in the NCBI database via BLAST-search [http://blast.ncbi.nlm.nih.gov/Blast.cgi, [73]]. An examination of five highly similar 16S rRNA sequences from the enrichment of heating water samples showed, that it mostly contained bacteria closely related to *Thermus scotoductus* [NCBI acc. no. EU330195.1; max. identity 97%, Expect (E)-value 0.0]. Twenty analyzed sequences revealed that the enrichment of soil and water samples from the Botanical Garden contained 70% bacteria belonging to the *Symbiobacterium* group with the highest similarity to *Symbiobacterium thermophilum* IAM 14863 (NCBI acc. no. NC_006177; 99% max. identity, E-value 0.0). The phylum *Bacillales* was represented by *Geobacillus*-species and uncultivated *Bacilli* to 25% (e. g. NCBI acc. no. AB548612.1; *Geobacillus debilis* gene for 16S rRNA, partial sequence, 99% max. identity, E-value 0.0), whereas 5% of the community comprised members of Clostridia (e. g. NCBI acc. no. FN667168.1; uncultured compost bacterium partial 16S rRNA gene, clone FS1689, 95% max. identity, E-value 0.0).

With the extracted DNA, large insert metagenomic libraries were constructed by using the cosmid vector pSuperCos and *E. coli* Epi100 as heterologous host. The library of the heating water enrichment culture comprised 576 clones, of which 28 analyzed clones had an average insert rate of 70%. The library of the soil and water samples enrichment consisted of 6,500 clones. The analysis of 87 clones showed an insert rate of 96%. Both libraries had an average insert size of 27.5 kb.

### Identification of the Lipolytic Genes lipS and lipT from Metagenomic DNAs

Screening of both libraries using a microtiter plate assay and *p*NP-dodecanoate as substrate identified four clones from the heating water enrichment library and six putative clones from the soil and water enrichment library that showed significant activities in these tests. The two most promising clones, one from each library, were characterized in detail. The positive clone from the heating water enrichment library was designated pCos6B1 and encoded a 27 kb insert. The clone from the soil and water samples enrichment library was designated pCos9D12 and encoded for a 26.5 kb insert. The cosmid clones pCos6B1 and pCos9D12 were subcloned in pTZ19R plasmids and transformed into *E. coli* DH5α. Sequencing of these subclones in combination with activity screening was pursued to identify the corresponding lipolytic genes. For pCos6B1, one subclone showed activity on TBT agar plates after incubation at 56°C and subsequently, a transposon mutagenesis was carried out and resulted in the identification of the corresponding ORF. The corresponding lipase gene on pCos9D12 was identified by sequencing in combination with primer walking. The ORFs linked to the lipolytic activities were designated *lipS* and *lipT* for the clones pCos9D12 and pCos6B1, respectively. The genes *lipS* and *lipT* encode putative proteins that consist of 280 and 331 amino acids, respectively. The translated gene sequences of *lipS* and *lipT* were compared with protein sequences deposited in the NCBI database by a BLASTX-search [73]. The BLASTX-search revealed their high similarities with genes annotated as putative esterases in known thermophilic microbes. The amino acid sequence of LipS shows 100% identity to a predicted *Symbiobacterium thermophilum* esterase (YP_075874) and LipT shows 97% identity to a predicted esterase from *Thermus scotoductus* (YP_004201971.1). LipT only showed low amino acid similarity to a previously described esterase EstTs1 of *Thermus scotoductus* [GenBank acc. no. ACS36170; 27.5% similarity according to a Needle (EMBOSS) alignment (http://www.ebi.ac.uk/Tools/psa/) [23]]. A common GXSXG motif that occurs in carboxylesterases and lipases was found in both enzymes. In LipS, the catalytic serine is embedded in a GLSMG motif, while LipT contained a GCSAG motif. Furthermore, sequence analyses with SignalP 4.0 [http://www.cbs.dtu.dk/services/SignalP/ [74]] indicated that *lipT* presumably encodes a secretion signal sequence with a cleavage site between Ala21 and Val22. For *lipS*, only a very low probability for a possible signal sequence was found with a hypothetical cleavage site between Ala17 and Gln18.

### Overexpression, Purification and Molecular Weight of LipS and LipT

Both genes *lipS* and *lipT* were cloned and overexpressed in order to verify the hydrolytic function of the corresponding enzymes and allow a biochemical characterization. Therefore, the genes were ligated into pET21a and transformed into *E. coli* BL21 (DE3). The recombinant enzymes contained a C-terminal His_6_-tag and were purified by Ni-NTA affinity chromatography under native conditions. LipS could be purified with 15.0 mg/g of cell pellet (wet weight). The maximum yield of LipT was 1.6 mg/g of pellet (data not shown). Thus, the protein yield after purification was overall better for LipS than for LipT (supplementary TABLE S5). The molecular weights of the proteins were verified by SDS-PAGE analysis under denaturing conditions. After Coomassie-staining, LipS was visible as a single band with a size of 31.7 kDa including the His_6_-tag (supplementary FIGURE S1A). LipT appeared to be at least a dimer, revealing a molecular weight of at least 78 kDa after incomplete denaturation (supplementary FIGURE S1B).However, a monomeric form of approx. 36 kDa corresponding with the calculated molecular weight was observed by a Western blot analysis using His_6_-tag specific antibodies (data not shown) and after extended heat denaturation of 30 min at 70°C (supplementary FIGURE S1A).

### Activity of LipS and LipT on Commercial pNP-ester Compounds

**Figure 1:**
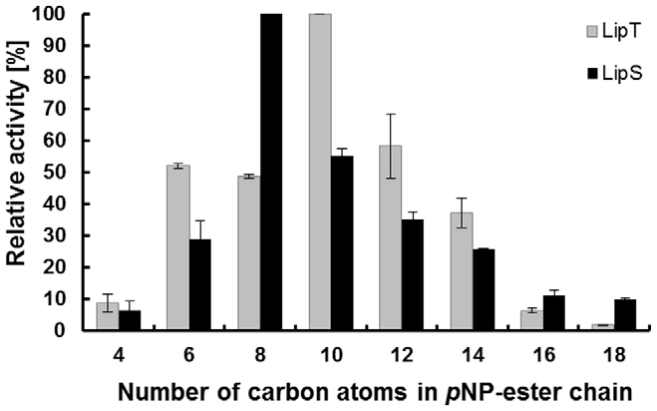
Substrate spectrum of LipS and LipT shown as relative activity on 4-nitrophenyl (*p*NP) esters with fatty acid chains of 4 to 18 C-atoms. Reactions were incubated at 70°C (LipS) or 75°C (LipT) with final substrate concentrations of 1 mM in potassium phosphate buffer (PB, 0.1 M, pH 8.0). Extinction was measured at 405 nm against an enzyme-free blank. Data are mean values of at least three independent measurements and bars indicate the standard deviation.

To characterize both enzymes, a substrate spectrum was recorded with *p*NP-esters which had an acyl chain length of 4 to 18 C-atoms. The highest activities were observed with *p*NP-octanoate in case of LipS and with *p*NP-decanoate in case of LipT (FIGURE 1). Both enzymes were most active between acyl-chain lengths of 6 to 14 C (25–58% of the maximum activity). Significantly lower activities were measured with short (C4) and long (C16 and C18) acyl chain lengths (FIGURE 1). Kinetic studies with the preferred substrates *p*NP-octanoate (LipS) and *p*NP-decanoate (LipT) disclosed significant differences between both enzymes (TABLE 1). LipS revealed a 20-fold higher specific activity compared to LipT and both enzymes differed in their K_m_ and k_cat_ values significantly.

**Table 1:** Biochemical parameters of recombinant LipT and LipS determined using 4-nitrophenol-decanoate (C10) for LipT and –octanoate (C8) for LipS.

Enzyme	U/mg	v_max_ (mol min^−1^)	K_m_ (mol l^−1^)	k_cat_ (min^−1^)	k_cat_/K_m_ (M^−1^ sec^−1^)
**LipT**	0.6	5.4 • 10^−8^	1.1 • 10^−3^	0.1	0.8
**LipS**	12.0	2.0 • 10^−7^	2.2 • 10^−3^	1.3	10.3

The measurements were performed at 75 and 70°C, respectively, in 0.1 M PB pH 8.0.

Data are mean values of three independent measurements.

### Temperature Optima, Thermostability and pH Dependent Activities of Recombinant LipS and LipT

**Figure 2:**
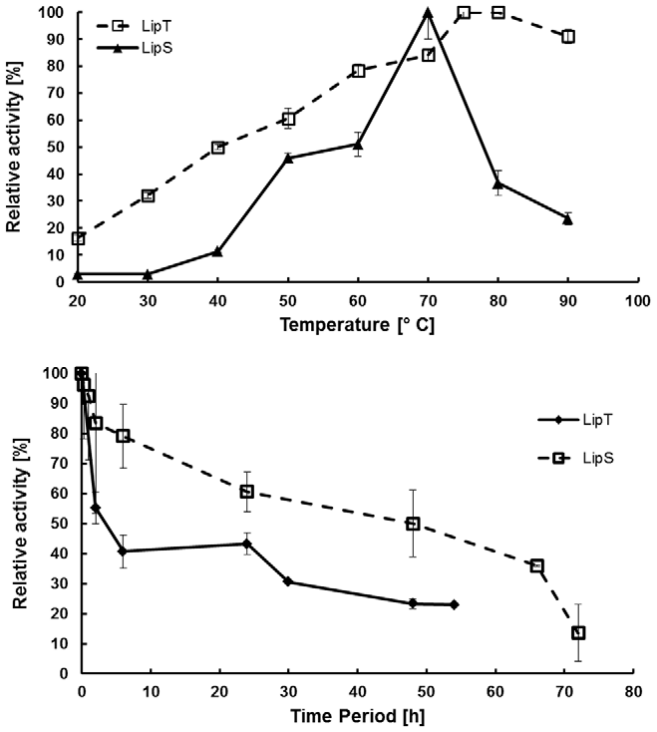
Temperature optimum (A) and thermal stability (B) of LipS and LipT. Data are mean values of at least three independent measurements and bars indicate the standard deviation. Temperature range and optimum of LipS and LipT were measured with *p*NP-dodecanoate at temperatures ranging from 20°C to 90°C for 10 min. Assays were performed by incubation of the enzymes at 70°C for up to 72 hours and by measuring residual activities with *p*NP-dodecanoate at 70°C (LipS) and 75°C (LipT).

Using 1 mM *p*NP-dodecanoate as substrate, the recombinant enzymes LipS and LipT revealed temperature optima of 70°C and 75°C, respectively. Interestingly, LipS was only weakly active at temperatures lower than 40°C, whereas LipT showed 50% of its activity at 40°C. Intriguingly, at 90°C, LipT still retained 91% of its maximum activity, LipS, however, only 23.5% (FIGURE 2A). To assess thermostability, both enzymes were incubated at elevated temperatures over extended time periods. After 48 h of incubation at 70°C, LipS revealed 50% residual activity; after 72 h, 13.6% of the activity could be measured (FIGURE 2B); incubated at 90°C, LipS still possessed 52% of its initial activity after 4 h of incubation. However, after 24 h, less than 1% of residual activity was measured at 90°C. LipT showed 43% residual activity after 24 h at 70°C and 23% after 52 h (FIGURE 2B). Incubation at 90°C for 24 h resulted in a residual activity of 22%. Altogether, these data suggest that both enzymes were thermostable.

LipS and LipT were most active at pH 8.0 when tested in 0.1 M PB and with 1 mM *p*NP-decanoate as substrate at their temperature optima. Below pH 8.0, activity was rapidly decreasing and at pH 6.0, only 11.4% (LipS) and 6.8% (LipT) residual activity was observed. Above pH 9.0, no significant activities were measured (data not shown).

### Activity of LipS and LipT in the Presence of Metal Ions, Inhibitors, Detergents and Solvents

To characterize the effects of metal ions, different ions (Ca^2+^, Co^2+^, Cu^2+^, Fe^3+^, Mg^2+^, Mn^2+^, Rb^2+^ and Zn^2+^) were added to the assays at 1 and 10 mM final concentrations. Activity was measured with *p*NP-dodecanoate and compared with a metal ion-free control. The activity of LipS as well as LipT´s activity decreased in the presence of most of these ions and no significantly stimulating effects indicating a cofactor-dependent activation were observed (supplementary FIGURE S2).

Furthermore, EDTA, DTT and PMSF were applied in final concentrations of 1 and 10 mM (FIGURE S3). EDTA decreased LipŚs activities at 1 mM to 74.1% and at 10 mM to 46.0% residual activity. The effects on LipT were less pronounced as it still revealed 98.0% residual activity at 1 mM EDTA and 65.7% at 10 mM EDTA. Incubation with 1 and 10 mM DTT resulted in a residual activity of LipS of 76.3% and 71.5%, respectively. LipT was not affected by the presence of 1 mM DTT and 85.4% of its activity remained in the presence of 10 mM DTT. PMSF did not show an effect on the activity of LipS in both concentrations of the inhibitor. LipT was inhibited by 10 mM PMSF to a residual activity of 49.0%, while lower concentrations of PMSF had no effect. SDS, Triton X-100 and Tween 80 were applied with 1 and 5% concentration (w/v, v/v) as detergents. With a final concentration of 1%, the substances lowered the activity of LipS only insignificantly. A concentration of 5% strongly decreased the activity to 0% (SDS), 14.3% (Triton X-100) and 20.1% (Tween 80). LipT was not even active in the presence of 1% SDS and was strongly affected by 1% Triton X-100. It revealed only 13.0% residual activity; and in the presence of 1% Tween 80, only 18.4% residual activity was observed. After incubation with 5% solutions of the two detergents Triton X-100 and Tween 80, LipT was almost completely inactivated (3.3% residual activity with Triton X-100; 0.3% residual activity with Tween 80).

The solvent stability of LipS and LipT was investigated in the presence of DMSO, isopropanol, methanol, DMF, acetone, acetonitrile and ethanol at concentrations of 10 and 30% (v/v) in 0.1 M PB pH 8.0 (TABLE S6). The presence of all solvents affected LipS. With 10% of solvent, residual activities between 67.9 and 27.7% were detected when compared to a solvent-free control, while 30% of solvent decreased the activities of LipS to 45.9–8.5%. The only exception was 30% of DMSO, where at least 92.9% of activities of both enzymes remained. Interestingly, LipT was much more stable in the presence of various solvents.

### Substrate Range and Enantioselectivity of LipS and LipT

LipS and LipT were tested for their hydrolytic activity on a wide range of substrates; among them achiral or racemic *p*NP-esters in a final concentration of 0.5 mM at 70°C (TABLE 2). LipS hydrolyzed 2-phenylpropanoate (0.42 U/mg), 3-phenylbutanoate (0.09 U/mg), cyclohexanoate (1.26 U/mg), 2-(3-benzoylphenyl) propanoate (0.62 U/mg), 2-naphthoate (0.06 U/mg), and 2-(4-isobutylphenyl)-N-propanamide ester (0.07 U/mg). The substrates 1-naphthoate and adamantanoate were, however, not converted by LipS. The substrate range of LipT was narrower in comparison, as it hydrolyzed 3-phenylbutanoate (0.03 U/mg), 2-(3-benzoylphenyl) propanoate (0.06 U/mg), 2-naphthoate (0.02 U/mg) and 2-(4-isobutylphenyl)-N-propanamide ester (0.08 U/mg). Interestingly, LipT hydrolyzed 1-naphtoate, even though with weak activity (0.01 U/mg). LipT did not cleave the ester bonds of 2-phenylpropanoate, cyclohexanoate and adamantanoate.

**Table 2:** Specific activity* (U/mg) of LipT and LipS on *p*NP esters.

*p*NP-Substrate		LipT	LipS	CalB	ROL
**Octanoate** 1)	/	+	+ +	+	+
**2-Phenylpropanoate** 2)	rac	−	+ +	n. d.	n. d.
**3-Phenylbutanoate** 2)	rac	+	+	n. d.	n. d.
**Cyclohexanoate** 2)	/	−	+ + + +	n. d.	n. d.
**2-(3-Benzoylphenyl) propanoate** 2)	rac	+	+ +	n. d.	n. d.
**2-Naphtoate** 2)	/	+	+	n. d.	n. d.
**1-Naphtoate** 2)	/	+	−	n. d.	n. d.
**Adamantanoate** 2)	/	−	−	n. d.	n. d.
**Methyldecanoate** 1)	(*S*)	+	+	+	+
	(*R*)	+	+ +	+	+
**2,3-Dihydro-1H-indene-1-carboxylate (indan acid ester)** 1)	(*S*)	+	+	+	−
	rac	+	+	+	+
	(*R*)	+	+ +	+	+
**Ibuprofen ester** 1)	(*S*)	+	−	+	−
	rac	+	+ +	+	+
**2-(4-isobutylphenyl)-N-(4-nitrophenyl) propanamide** **(Ibuprofen amide ester)** 2)	rac	+	+	n. d.	n. d.
**Naproxen ester** 1)	(*S*)	+	+	+	+
	rac	+	+	+	+
	(*R*)	+	+ +	+	+

CalB (purchased from Sigma-Aldrich, Buchs, Switzerland) and ROL (purchased from Fluka/Sigma-Aldrich, Buchs, Switzerland) were used as references.

The extinction was measured spectrophotometrically against an enzyme-free blank with

1) 0.33 mM substrate solution (final concentration in 0.05 M Soerensen buffer pH 8.0 containing 0.1% gum arabic, 5 mM sodium deoxycholate and 10% DMSO) after incubation at 60°C (CalB) or 65°C (LipT, LipS, ROL) at 410 nm (ε = 7,392 M^−1^ cm^−1^).

2) 0.5 mM substrate solution (final concentration in 0.1 M PB pH 8.0) after incubation at 70°C at 405 nm (ε = 19,454 M^−1^ cm^−1^).

* Specific activity: n. d., not determined; −, no detectable activity or <0.01 U/mg; +, 0.01–0.30 U/mg; + +, 0.31–0.60 U/mg; + + +, 0.61–0.90 U/mg; + + + +, 0.91–1.26 U/mg. Specific activities of CalB and ROL refer to the dry-weight of the lyophilisate. Data are mean values of three independent measurements.

**Figure 3:**
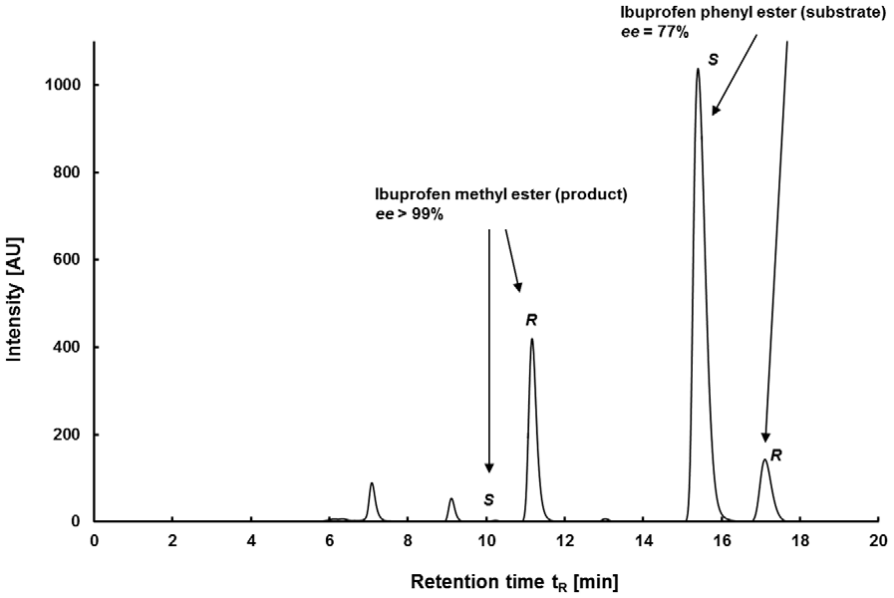
HPLC-MS measurement of LipS catalyzing (*R*)-selectively the hydrolysis of ibuprofen phenyl ester. The products of the reaction were converted to the corresponding methyl esters for measurement.

The stereoselectivity of LipS and LipT were assayed on chiral *p*NP-esters namely (*S*)-/(*R*)-2-methyldecanoic acid ester, (*S*)-/rac-/(*R*)-2,3-dihydro-1*H*-indene-1-carboxylate (“indancarboxylic acid ester”), (*S*)-/rac-ibuprofen-ester and (*S*)-/rac-/(*R*)-naproxen-ester (TABLE 2). Reactions with *p*NP-esters as substrates were measured after incubation at 60°C and 65°C. These relatively mild temperatures were chosen to avoid autohydrolysis that readily occurs at higher temperatures. In comparison, the commercial enzymes CalB and ROL (*Rhizopus oryzae* lipase) were tested at the same temperatures. CalB and LipT did not show stereoselectivity. ROL showed a preference for the (*R*)-enantiomer of indancarboxylic acid ester and ibuprofen ester. The highest activity of all enzymes at this temperature was observed with LipS and it also revealed the most distinct enantioselectivity, as it was more active on the (*R*)-enantiomers of the different substrates. LipS favored the (*R*)-enantiomers of 2-methyldecanoic acid ester (*E* = 8), indancarboxylic acid ester (*E* = 12) and naproxen-ester (*E* = 9) [75]. It, however, revealed only very poor activities on the (*S*)-ibuprofen ester (TABLE 2). This result was verified by HPLC analysis. LipS preferred the (*R*)-enantiomer of ibuprofen *p*NP-ester with an *ee* of >>59% for the product and ∼90% for the remaining substrate (*E* = 11, conversion 60%). The stereoselectivity of LipS was even higher on ibuprofen phenyl ester, where an *ee* of 99% was detected for the product and 81% for the substrate at 45% conversion for the phenyl ester which leads to an *E*-value >>100 (FIGURE 3).

### Activity of LipS on Tri- and Polyglycerides

Furthermore, we assayed the activities of LipS and LipT on tri- and polyglycerides. LipT did not reveal significant activities in the titration assays using tributyrin, triolein and polyglycerol-3-laurate as substrates. However, LipS had a specific activity of 0.14 U/mg at 60°C using 50 mM tributyrin. The activity was higher with 50 mM triolein (0.20 U/mg); and LipS revealed 0.61 U/mg on a 7.5% emulsion of polyglycerol-3-laurate.

### Kinetic Resolution of Acetates of Secondary Alcohols

In addition, the enantioselective hydrolysis of four acetates of secondary alcohols was investigated using LipS. Whereas the hydrolysis of 1-phenyl-1-propyl acetate and 1-phenyl-1-ethyl acetate proceeded with low enantioselectivity (*E* = 3–4), excellent selectivity of LipS was observed for 1-phenyl-2-butyl acetate and 1-phenyl-2-pentyl acetate. In both cases, the corresponding chiral (*R*)-alcohols were obtained with >96% *ee* at approx. 50% conversion. This suggests that selectivity of LipS towards secondary alcohols is higher if the chiral center is not adjacent to the aromatic ring, but a further CH_2_-group away to enable high discrimination of the two enantiomers.

### Esterification by LipS

**Figure 4:**
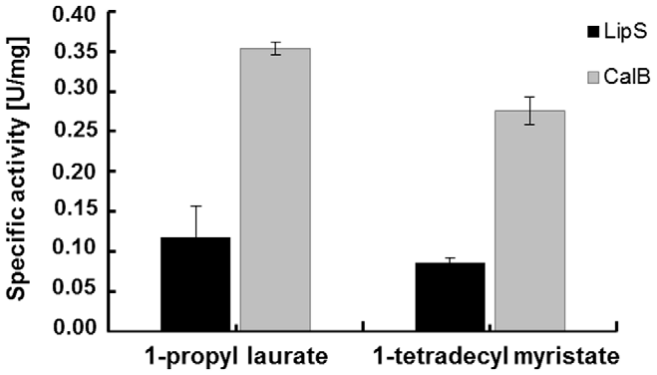
Esterification reactions between 1-propanol and lauric acid (20 mmol each) as well as 1-tetradecanol and myristic acid (15 mmol each). Synthesis reactions were catalyzed by LipS and CalB (purchased from Sigma-Aldrich, Buchs, Switzerland) under solvent-free conditions at 70°C. Specific activities of LipS and CalB refer to the dry-weights of the lyophilisates. Data are mean values of at least three independent measurements and bars indicate the standard deviation.

To further study the esterification of LipS, the enzyme activity was assayed in the propyl laurate assay and benchmarked with CalB as a control. At 70°C, the esterification reactions between 1-propanol and decanoic acid as well as 1-tetradecanol and myristic acid were catalyzed by 15 mg of lyophilized LipS and CalB. After 48 h, the formation of 1-propyl laurate was catalyzed by LipS (0.12 U/mg) and CalB (0.35 U/mg). The synthesis of 1-tetradecyl myristate also took place with LipS (0.09 U/mg) and CalB (0.28 U/mg) (FIGURE 4).

### Crystallographic Analysis of LipS

A variety of constructs were expressed and purified for crystallization experiments as described earlier [67]. The initial construct LipS-H6 in SG P4_2_2_1_2 diffracted X-ray radiation to 2.80 Å resolution, while a second construct, LipS-WT, diffracted X-ray radiation to 1.99 Å resolution. Both constructs contain the native N-termini which are disordered until about residue 35.

**Figure 5:**
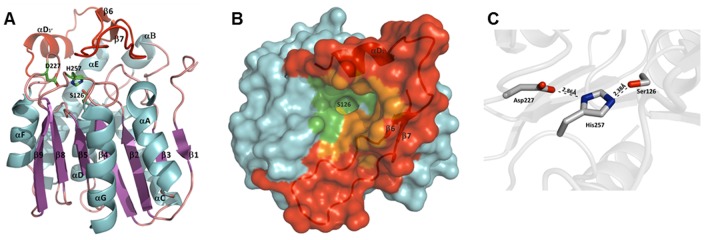
Protein structure of LipS. **A)** Ribbon representation of the LipS monomer colored according to secondary structure elements. The inserted lid-domain is indicated in red. The catalytic triad residues Ser126, His257 and Asp227 are shown as stick representation. **B)** Surface representation of the LipS monomer with the lid-domain (β6, β7, αD_1_′) shown as a cartoon representation in red. The active site S126 (in yellow) is completely occluded from the bulk solvent and only accessible through a narrow tunnel. The active site pocket identified by CASTp server is colored in green. Amino acids building a pocket as part of the inserted domain are shown in orange. **C)** The catalytic triad residues of LipS are properly placed to establish hydrogen bonds.

LipS displayed a dimeric character during purification by size exclusion chromatography. Consistent with this observation, the asymmetric units of LipS in SG P4 and P4_2_2_1_2 contain one and two identical dimers, respectively. Analysis of the LipS-WT and LipS-H6 interfaces with Protein Interfaces, Surfaces and Assemblies (PISA) server [76] calculates the buried area between two protein molecules and based on solvatation energy (ΔG) gained upon assembly formation, it calculates a complexation significance score (CSS). The analysis confirms that the observed interactions are of biological significance (CSS = 0.69; ΔG = −20.5), because the CSS is expressed on a scale from 0, for non-significant interface, to 1, for significant interface. The dimer interface covers 1245 Å^2^ (12.2% of the total surface of a monomer) of accessible surface area per monomer. It is primarily formed by the short helical segment αD_1′_ at the N-terminal part of the insertion and the long helix αD (FIGURE 5A). Several hydrogen bonds and salt bridges involving Q138, R154, A162, T203 and E209, in addition to numerous hydrophobic contacts, stabilize the dimer interface.

LipS assumes the fold of a classical α/β hydrolase [77]. Members of this fold family accommodate a wide variety of enzymatic activities, including lipases, esterases, peroxidases, dehalogenases and epoxide hydrolases [78]. It consists of a central β-sheet made of six parallel β-strands (β2, β3, β4, β5, β8, β9) and one antiparallel β-strand (β1). Hence, the central β-sheet is missing the first β-strand of the canonical α/β hydrolase architecture so that it consists of 7 instead of 8 strands. The central β-sheet is sandwiched by helices αA and αG on one side and helices αB through αF on the opposite side (FIGURE 5A). The active site of LipS is formed by the catalytic triad S126, D227 and H257 with the catalytic serine located at the sharp γ-turn between β4 and αD (FIGURE 5C). The position of the catalytic triad and the oxyanion hole (F58 and M127) at conserved topological sites clearly designates this newly characterized enzyme as a hydrolase.

After refinement, the active site of LipS-WT in SG P4 contained strong residual density immediately adjacent to S126 and H257. This density was interpreted with spermidine (FIGURE S4A, B). Spermidine was used as an additive to improve crystal quality and was subsequently shown to inhibit the activity of LipS with its substrate *p*NP-decanoate in concentration dependent manner (FIGURE S4C). The terminal amino-group of spermidine comes remarkably close to both S126 and H257 when the secondary amid group interacts with D187, which lines the end of the active site cavity. Thus, it is likely that spermidine mimics substrate bound in the active site (FIGURE S4D).

**Figure 6:**
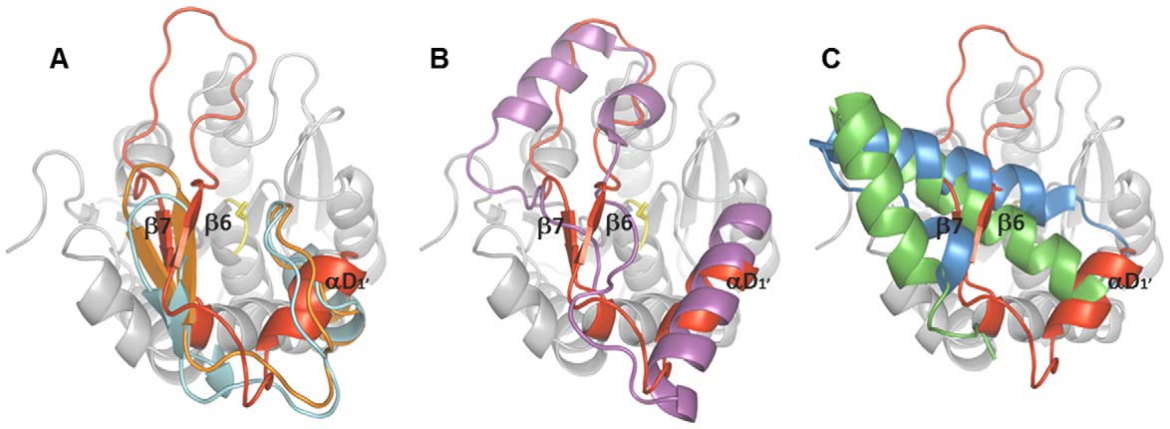
Topology of the inserted domains of α/β-hydrolases. Superimposition of the inserted domain of LipS (in red) with **A)** Est1E (2WTM, orange) and LJ0536 (3PF8, turquoise), **B)** human MGL (3PE6, purple) and **C)** EstD (3DKR, blue) and Est30 (1TQH, green). The core structure of LipS is indicated in grey and catalytic S126 in yellow. The core structures of LipS homologues are not shown for simplicity.

We reasoned that comparing 3D structures may reveal biologically interesting similarities that were not detectable by comparing amino acid sequences. Therefore, the comparison of LipS with related 3D structures was performed using DALI server [79]. The structurally most closely related enzymes were esterases, Est30 from *Bacillus stearothermophilus* [1TQH, *Z*-score 30.7, RMSD 1.8, [80]], EstD from *Lactobacillus rhamnosus* [3DKR, *Z*-score 28.7, RMSD 1.8 [81]], Est1E from *Butyrivibrio proteoclasticus* [2WTM, *Z*-score 24.0, RMSD 2.4 [82]] and human mono-glyceride lipase [3PE6, *Z*-score 28.1, RMSD 2.2 [83], FIGURE S5]. Structural superimposition of LipS with these four enzymes revealed notable similarity of their overall structures which all resemble the α/β-hydrolase fold (FIGURE 6). The core of the α/β-hydrolase fold, the central β-sheet and flanking α-helices, was highly similar between them (RMSD 1.2 Å to 1.8 Å) contrary to the 40 amino acid large subdomain (E156 to V195) inserted between β5 and αE of LipS. This subdomain of LipS is surface exposed and folds into a short helix αD_1_′ and two short antiparallel β-strands, β6 and β7. Among above listed structural homologues, only Est1E has a mixed α/β secondary structure topology similar to LipS. The inserted subdomains of Est30, EstD and human MGL all have a α-helical topology which differs from the topology of LipS (FIGURE 6B, C). Recently, the topology of an inserted subdomain similar to the one from Est1E was recognised in the cinnamoyl esterase LJ0536 from *Lactobacillus johnsonii* [84], which is apparently not deposited in the DALI database and thus, it was not detected as a structural homologue of LipS. The core structure of the α/β-fold of LJ0536 resembles the structure of LipS like the other above mentioned cores of the LipS homologues.

Superimposition of LipS with its homologues and inspection of inserted domains revealed similarity of LipS with Est1E, LJ0536 but also with evolutionarily distant human MGL. Based on the presence of β-strands (β6, β7) in the inserted domain of LipS, which are indeed structurally equivalent with β9, β10 of LJ0536 and β9, β10 of Est1E (FIGURE 6A), it seems that the inserted domains of these three enzymes are structurally related. It is noteworthy, that shifting of this β9/β10 hairpin of Est1E was proposed to lead to the formation of a substrate binding hydrophobic pocket [84]. However, notable differences between inserted domains of these three enzymes were observed. Thus, the loop connecting β6 and β7 in LipS is 17 amino acids long compared to 3 and 4 in Est1E and LJ0536, respectively. Furthermore, the short helix αD_1_′ of LipS did not superimpose with any of the α-helices in Est1E and LJ0536. Additionally, the second short β-hairpins of Est1E (β7/β8) and LJ0536 (β7/β8) are absent in LipS. Although the secondary structure topology of the inserted domain of human MGL (α/α/α-fold) is diverse to the one of LipS (β/β/α-fold), it resembles its eukaryotic counterpart in MGL more closely than in Est1E and LJ0536 (FIGURE 6B). The αD_1_′ of LipS superimposed well with α4 of human MGL, although α4 is 10 residues longer than αD_1_′. Interestingly, an important biological function of the hydrophobic α4 for docking of human MGL onto membranes in order to gain access to the lipid substrates was suggested [85]. The part of LipŚs inserted domain containing two β-strands β6 and β7 and a loop connecting them superimposed well with the region in human MGL ranging from 174 to 206, made by loops and the α5 and 3/10-helix, which is identical in its size to the LipS motif. Our results indicate that biologically important structural features of both prokaryotic and eukaryotic lipases are unified in the inserted subdomain of LipS and thus, LipS might represent an enzyme which is on evolutionary scale placed between eukaryotic and prokaryotic lipases.

**Figure 7:**
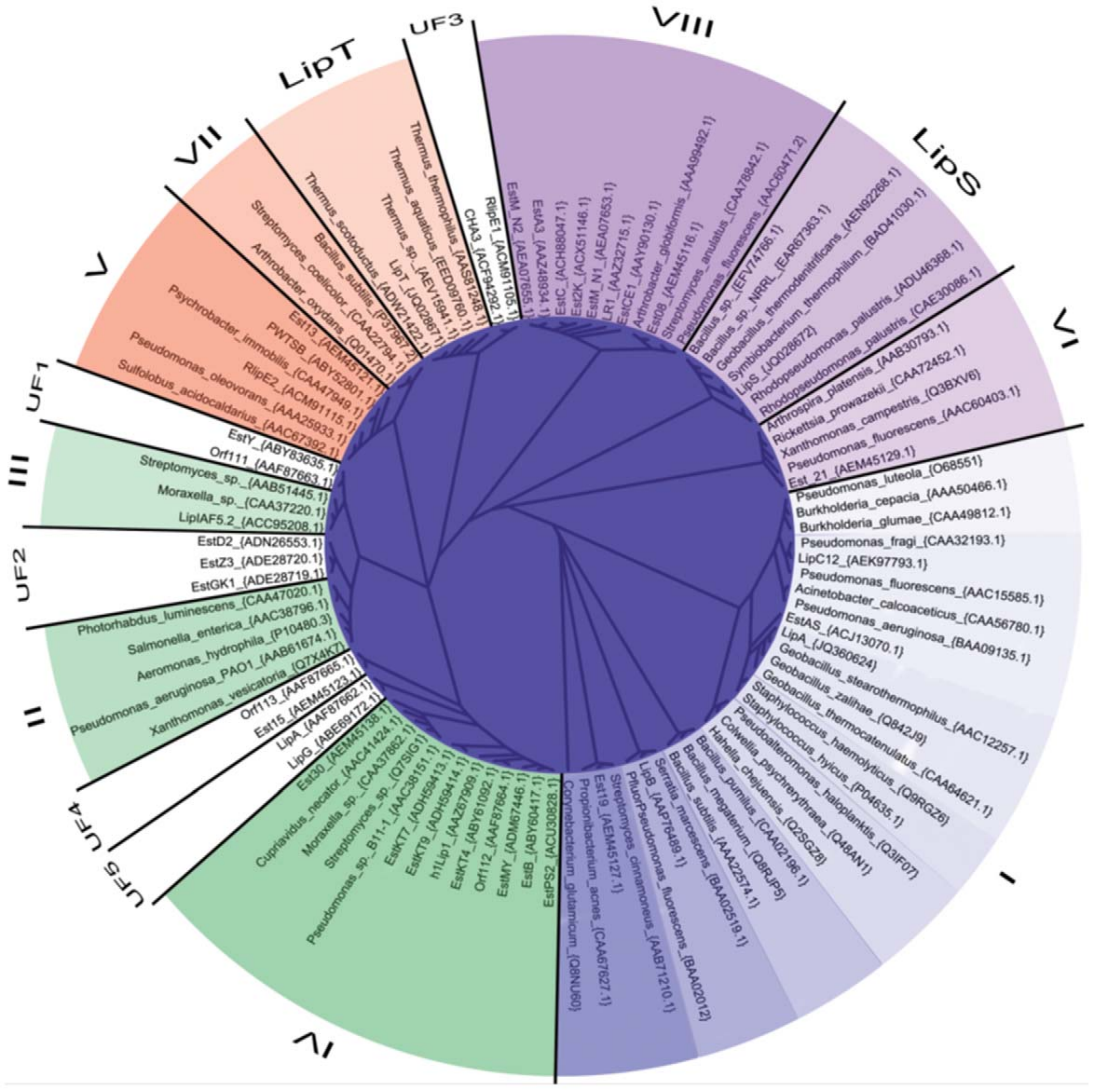
Phylogenetic tree illustrating the sorting of 40 metagenome derived lipase/esterase sequences into the eight known lipase/esterase families [**61**]. The eight families are color coded and labeled with the respective family name (LipS, LipT) or number (I-VIII). The five subfamilies containing the 11 unassignable metagenome lipase/esterase sequences are shown in white and are labeled with the respective family name (UF1-UF5). For the reference sequences, the full organism name as well as the accession number is given at the respective clade. Metagenome sequences are labeled with their protein name and accession number, respectively.

Insertions of different lengths and conformations in other α/β-hydrolases at that location [86] suggest their evolutionarily importance for distinct biological functions of the enzymes. These inserted subdomains have dual biological function, a) as a lid which, in dynamic process, opens and closes the active site for exposure to the solvents and substrates and b) as a motif that shapes the active site for accommodation of appropriate substrates. Indeed, the flexible inserted domains serving as a lid were suggested for Est1E and human MGL but not for LJ0536, which adopts the same conformation in absence and presence of a ligand bound in the active site. The conformations of the inserted domains in all four LipS molecules found in one asymmetric unit were identical. Furthermore, data that the inserted domain assumes the same conformation with and without bound spermidine in structures of LipS suggests that this is rather a rigid structure.

**Table 3:** Activities of LipS and LipT in comparison with other characterized and published bacterial thermostable lipases.

Source	T_opt_ [°C]	*p*NP-substrate	Specific activity [U/mg]	Reference
**LipS (metagenomic)**	70	C8	12.03	This study
		C10	6.04	
**LipT (metagenomic)**	75	C10	0.6	This study
**Est53, ** ***T. maritima***	60	C12	13.0	[107]
**LipA, ** ***T. lipolytica***	96	C12	12.4	[24]
**LipB, ** ***T. lipolytica***	96	C12	13.3	[24]
**LipTth, ** ***T. thermohydrosulfuricus***	75	C16	12.15	[15]

Only lipases with temperature optima of ≥60°C and activity on *p*NP-substrates with ≥8 C atoms as acyl residue were considered.

Analysis of the cavities on LipŚs surface using the Computed Atlas of Surface Topography of proteins (CASTp) server [87] revealed only one pocket in vicinity of the catalytic S126 (FIGURE 5B). This pocket with an area of 546.1 Å^2^ was defined as an active site pocket. 30 amino acids, 20 hydrophobic and 10 hydrophilic (TABLE S7), contribute in formation of the active site pocket, thus providing amphipathic environment for substrate binding. Similarly to human MGL, the binding pocket of LipS is occluded with only narrow and restricted opening to the bulk solvent (FIGURE 5B). Human MGL, Est1E and LJ0536, although similar to LipS, have their active site much more exposed to the solvent compared with LipS. Thirteen amino acids of the inserted domain (TABLE S7) contribute at the same time in formation of the active site pocket of LipS (FIGURE 5B). Similar with other LipS homologues, the inserted domain of LipS shapes the catalytic pocket of LipS. Not surprisingly, mutations of inserted domain of Est1E have affected its substrate specificity [82]. Therefore, we would like to propose that the novel fold of the inserted domain of LipS, at the frontier between eukaryotic and prokaryotic lipases, could be essential for its selectivity in hydrolysis of a range of complex substrates listed in TABLE 2.

### Classification of LipS and LipT

Using modern alignment methods, we tried to sort 40 metagenome derived lipase/esterase sequences into the 8 known bacterial lipase/esterase families [61]. The sequences were grouped by aligning them manually to a subset of sequences representing the respective family. The quality of the independently calculated alignments is reasonably good, as judged from visual inspection, conservation of key amino acids [61] and T-coffee alignment scores. Nevertheless, the low sequence conservation between the eight families did not directly allow the construction of a meaningful alignment for the full dataset. Therefore, the seed alignment had to be constructed by assembling the sub-alignments in into a full dataset. Thus, the presented tree (FIGURE 7) serves solely as an illustration for the assignment of the metagenome derived sequences to the eight known bacterial lipase/esterase families, but does not allow any conclusions with respect to the relationship between the respective families. Using this alignment strategy, 11 out of 40 metagenome derived sequences could not be assigned to any of the eight known and established families. Likewise, LipS and LipT together with a set of homologous sequences could not be assigned unequivocally based on sequence similarity (FIGURE 7). This sequence comparison thus suggests that they are both part of novel lipase families without distinctive similarity to any of the known eight bacterial lipase/esterase families [61].

## Discussion

We have isolated two novel lipase genes from metagenomic samples by a combined enrichment and direct cloning approach. Two different enrichment cultures were set up and 65 as well as 75°C were chosen as incubation temperatures in order to cover a broader spectrum of thermophilic organisms. Although the combined enrichment and metagenome technology applied significantly reduces the overall biodiversity in the environmental sample, it has been successfully applied by our lab and others to identify numerous useful biocatalyst genes from metagenomic samples [88]–[91]. In one such study, even a moderately thermostable metagenomic lipase was identified [92].

The two lipase genes identified in this work shared high similarities with already known genes in the databases. LipS was similar to a predicted but not characterized esterase from the compost bacterium *Symbiobacterium thermophilum* and LipT was similar to a predicted esterase from *Thermus scotoductus*. Interestingly, *Symbiobacterium thermophilum* is supposed to have the highest content of horizontally acquired genes among all bacteria known so far [93]. Of its protein coding genes, 17.7% originate from Bacilli and 36.9% from Clostridia. *S. thermophilum* can be isolated from enrichment cultures using compost or soil as inoculum [94]. It grows at an optimum temperature ranging from 45 to 65°C. However, it is uncultivable as a single species and it relies on commensalism [94]. Up to date only a single genome has been published having a size of 3.6 Mb [95].

While more than 100 strains of *Thermus* have been reported [96] only very few *T. scotoductus* isolates are known. *T. scotoductus* has been isolated from thermal springs but it can also be found in man-made sources such as gold mines [97], [98]. Only recently, the first genome of a *T. scotoductus* strain, i.e. SA-01, was established and revealed a genome size of 2.4 Mb [99]. *T. scotoductus* usually grows at temperatures between 65 and 70°C [100], [101], while other *Thermus* species have their optimal growth temperature between 62°C and 75°C [96]. Interestingly, the isolation of *T. thermophilus* from heating water systems has been reported [102]. Within this context it is notable that heating water systems harbor obviously rather diverse microbial communities [103], [104].

The classification of LipS and LipT into one of the lipase and carboxylesterase families according to Arpigny and Jaeger [61] was not possible. Both enzymes are thus most likely members of novel lipase families (FIGURE 7) which presumably contain other lipase-members derived from cultivated thermophilic microbes as well.

It is noteworthy, that LipS and LipT represent the first metagenome-derived lipases that reveal a temperature optimum of ≥70°C. Since both enzymes, however, were derived from metagenomes, we can only speculate about their native substrates and functions within the cells.

We have characterized the substrate spectra of both enzymes in great detail using a range of industry-relevant substrates. Both LipT and LipS showed a clear preference for *p*NP-esters with long chained fatty acid residues (>C8), their temperature optima were at 70 and 75°C and both enzymes showed a high thermal stability at 70 as well as at 90°C. Both LipS and LipT do not require cofactors and are stable against most detergents, solvents and even enzyme inhibitors. Especially the substrate range of LipS is not limited to *p*NP-esters with simple fatty acid residues. It is also able to hydrolyze sterically more complex substrates with phenolic or cyclohexanoic residues. LipS showed a high (*R*)-selectivity for ibuprofen, naproxen, methyl decanoic acid and indancarboxylic acid ester, which can be useful for the production of chiral pharmaceuticals. Ibuprofen, for example, is physiologically active as (*S*)-enantiomer [105]. LipS could be applied in the dynamic kinetic resolution of the racemate by hydrolyzing remaining (*R*)-enantiomers in order to obtain an enantiopure product [106]. With respect to the catalytic activities of both enzymes, they showed comparable or better activities than activities published for other thermostable bacterial lipases [[15], [24], [107], TABLE 3]. Further, in small-scale experiments, esterification reactions were catalyzed by LipS with the result that 1-propyl laurate as well as 1-tetradecyl myristate were produced with non-immobilized enzyme. The observed activities were comparable to those observed for *Candida antarctica* lipase CalB. Altogether, these data suggest that LipS and LipT are very promising enzyme candidates for biotechnological applications at elevated temperatures.

The structure of LipS was solved for two different constructs which crystallized in two different space groups. In the higher resolution SG P4, there is clear additional electron density immediately adjacent to the catalytic S126 and H257 (FIGURE S4A, B). In LipS in SG P4_2_2_1_2, however, there is no such electron density. This excludes the possibility that the observed density results from expression in *E. coli*. We interpreted this residual density with spermidine, which was added during crystallization of LipS in SG P4 but not in SG P4_2_2_1_2. At this point, there is no indication that spermidine is part of any native substrate of LipS. Identical conformations of the lid-domain, which covers the active site, in the absence and presence of a ligand, indicate that this domain may not undergo conformational rearrangements during the catalytic cycle. Concerning Est1E and LJ0536, it was shown that the lid is flexible [82], [84]. The closed lid-conformation resulted in an occluded binding pocket, which most likely only leaves a narrow and restricted opening to the bulk solvent. Occlusion of the binding pocket in human monoglyceride lipase MGL, together with structural rearrangements of the hydrophobic lid in MGL, are thought to support the extraction of its substrate 2-arachidonoyl glycerol from the membrane by providing an accommodating environment [83]. While the precise cellular substrate of LipS is unknown, its surface does not indicate increased hydrophobicity around the binding pocket. A closed, water secluded active site may therefore provide a protective environment to prevent spurious hydrolysis of its substrate at the elevated level of its optimal temperature range.

In summary, LipS and LipT are both very interesting and promising enzymes with a high potential for downstream biotechnological applications. This was confirmed by their extensive biochemical characterization and in the case of LipS this was supported by the structural data. The sequence and structural characterization clearly suggests that both enzymes increase the diversity of known esterase and lipase families.

## Supporting Information

Figure S1 **15% SDS-PAGE of recombinant and purified LipS and LipT.** Asterisks indicate the corresponding protein bands after His6-tag affinity chromatography. 15 µg of protein from the crude cell extracts or from the purified proteins were loaded and electrophoresed. **A)** 1, 4: crude cell extract; 2, 3 purified protein after extended heat treatment (30 min at 70°C and 5 min 95°C). **B)** Purified LipT after incomplete heat-denaturation (5 min 95°C).
(TIF)

Figure S2 **Effect of metal ions applied in 1 and 10 mM concentration on LipT and LipS.** Residual activity of the enzymes was measured with *p*NP- dodecanoate at 75°C (LipT) and 70°C (LipS). Compared with the control without metal ions, none of the cations showed positive effects significant enough for being considered as cofactor. Data are mean values of at least three independent measurements and bars indicate the standard deviation.
(TIF)

Figure S3 **Effects of 1 and 10 mM EDTA, DTT and PMSF on the activity of LipS and LipT.** The residual activity was measured at 70°C (LipS) and 75°C (LipT) using *p*NP-substrates.
(TIF)

Figure S4 **Effect of spermidine on the active site of LipS. A)** Electron density maps (blue) around S126, H257 and spermidine. The additional density linking both residues and extending further towards the active site cavity was interpreted as spermidine. **B)** The spermidine moiety, shown as a space model colored in grey, is located in the active site cavity of LipS in vicinity of catalytic S126 and H257 indicated as sticks model. **C)** Spermidine inhibits LipS activity at concentrations of 3 and 5 mM compared to a control without added spermidine. Enzyme activity was determined after 5 min preincubation with spermidine using *p*NP-decanoate as substrate and incubation at 70°C for 10 min. **D)** Spermidine displays similarity with LipŚs substrate *p*NP-decanoate.
(TIF)

Figure S5 **Structure based sequence alignment of LipS with its homologues.** It revealed very low structural similarity in the region of the inserted domain, which is indicated by bold letters in LipS. 2WTM, Est1E form *Butyrivibrio proteoclasticus* [82]; 3PF8, LJ0536 from *Lactobacillus johnsonii* [84]; 3PE6, monoglyceride lipase (MGL) from *Homo sapiens* [83]; 1TQH, Est30 from *Geobacillus stearothermophilus* [80]; 3DKR, esterase D from *Lactobacillus rhamnosus* [81]. In the top line, secondary structure elements of LipS are shown with the inserted domain (αD_1′_, β6 and β7) colored in gray. Identical and similar amino acids conserved in at least four structures were shaded in black and gray respectively. Catalytic triad residues of LipS are indicated in bold and yellow. Residues which are not seen in the structures are shown as small letters.
(TIF)

Table S1 **Bacterial strains and plasmids used in this work.**
(DOCX)

Table S2 **Amino acid sequences of members from the eight major lipase and esterase families** [**61**] **received from GenBank together with members of the new LipS and LipT groups.**
(DOCX)

Table S3 **Metagenomic esterases and lipases from uncultured organisms grouped into existing families** [**61**] **.** The classification is based on alignment scores with members of family I-VIII or unknown families (UF). **UF1-5: Unknown Families of metagenomic esterases/lipases:** Sequences were grouped together into one family if at least two unclassified sequences shared significant similarity and sufficiently high T-COFFEE alignment scores. All sequences considered here as unclassified cannot be unequivocally grouped into any of the known other eight lipase/esterase families. *:I/S/G: Identical/Similar/Gapped amino acid positions in the respective alignment. Sequence identity/similarity is given with respect to one of the sequences of a known organism. Identity/similarity can be higher to metagenomic sequences in the subfamily.
(DOCX)

Table S4 **Refinement and quality statistics of the crystallized constructs LipS-H6 and LipS-WT.**
(DOCX)

Table S5 **Purification table of the proteins LipS and LipT.** (1) Crude cell extract; (2) heat denaturation at 70°C for 30 min; (3) immobilized metal ion affinity chromatography with Ni-ions. In case of LipT, the elution fractions have been combined and concentrated to a residual volume of 0.4 ml in a centrifuge (Vivaspin20, MWCO 10,000; Sartorius Stedim Biotech GmbH, Göttingen, Germany; Centrifuge 5804R, rotor A-4-44, Eppendorf, Hamburg, Germany). Activity was measured using 0.5 mM *p*NP-octanoate at 70°C (LipS) or 0.5 mM *p*NP-decanoate at 75°C (LipT).
(DOCX)

Table S6 **Residual activities of LipS and LipT in the presence of organic solvents.** The enzymes were incubated for 1 h at room temperature with the solvents diluted in 0.1 M PB pH 8.0 before *p*NP-dodecanoate was added as substrate. After incubation for 10 min at 70°C (LipS) and 75°C (LipT), the reaction was measured in a photometer at 405 nm against an enzyme-free blank containing the respective solvent and concentration. Data are mean values of at least three independent measurements; ± indicates the standard deviation.
(DOCX)

Table S7 **Amino acids and atoms building the active site pocket of LipS.** Amino acids belonging to the inserted domain are indicated in bold.
(DOCX)

## References

[1] Jaeger KE, Reetz MT (1998) Microbial lipases form versatile tools for biotechnology. Trends Biotechnol 16: 396–403. 9744114 10.1016/s0167-7799(98)01195-0

[2] Jaeger KE, Dijkstra BW, Reetz MT (1999) Bacterial biocatalysts: molecular biology, three-dimensional structures, and biotechnological applications of lipases. Annu Rev Microbiol 53: 315–351. 10547694 10.1146/annurev.micro.53.1.315

[3] Jaeger KE, Eggert T (2002) Lipases for biotechnology. Curr Opin Biotechnol 13: 390–397. 12323363 10.1016/s0958-1669(02)00341-5

[4] Reetz MT (2002) Lipases as practical biocatalysts. Curr Opin Chem Biol 6: 145–150. 12038997 10.1016/s1367-5931(02)00297-1

[5] Liese A, Seelbach K, Wandrey C (2006) Industrial Biotransformations; Liese A, Seelbach K, Wandrey C, editors. Weinheim: WILEY-VCH Verlag GmbH & Co. KGaA.

[6] Turner P, Mamo G, Karlsson EN (2007) Potential and utilization of thermophiles and thermostable enzymes in biorefining. Microb Cell Fact 6: 9. 17359551 10.1186/1475-2859-6-9 PMC1851020

[7] Yeoman CJ, Han Y, Dodd D, Schroeder CM, Mackie RI, et al (2010) Thermostable enzymes as biocatalysts in the biofuel industry. Adv Appl Microbiol 70: 1–55. 20359453 10.1016/S0065-2164(10)70001-0 PMC4561533

[8] Bruins ME, Janssen AE, Boom RM (2001) Thermozymes and their applications: a review of recent literature and patents. Appl Biochem Biotechnol 90: 155–186. 11297390 10.1385/abab:90:2:155

[9] Vieille C, Burdette DS, Zeikus JG (1996) Thermozymes. Biotechnol Annu Rev 2: 1–83. 9704095 10.1016/s1387-2656(08)70006-1

[10] Li WF, Zhou XX, Lu P (2005) Structural features of thermozymes. Biotechnol Adv 23: 271–281. 15848038 10.1016/j.biotechadv.2005.01.002

[11] Dalhus B, Saarinen M, Sauer UH, Eklund P, Johansson K, et al (2002) Structural basis for thermophilic protein stability: structures of thermophilic and mesophilic malate dehydrogenases. J Mol Biol 318: 707–721. 12054817 10.1016/S0022-2836(02)00050-5

[12] Ebrahimi M, Lakizadeh A, Agha-Golzadeh P, Ebrahimie E, Ebrahimi M (2011) Prediction of thermostability from amino acid attributes by combination of clustering with attribute weighting: a new vista in engineering enzymes. PloS one 6: e23146. 21853079 10.1371/journal.pone.0023146 PMC3154288

[13] Hough DW, Danson MJ (1999) Extremozymes. Curr Opin Chem Biol 3: 39–46. 10021406 10.1016/s1367-5931(99)80008-8

[14] Schiraldi C, De Rosa M (2002) The production of biocatalysts and biomolecules from extremophiles. Trends Biotechnol 20: 515–521. 12443873 10.1016/s0167-7799(02)02073-5

[15] Royter M, Schmidt M, Elend C, Hobenreich H, Schafer T, et al (2009) Thermostable lipases from the extreme thermophilic anaerobic bacteria *Thermoanaerobacter thermohydrosulfuricus* SOL1 and *Caldanaerobacter subterraneus* subsp. tengcongensis. Extremophiles : life under extreme conditions 13: 769–783. 19579003 10.1007/s00792-009-0265-z PMC2757599

[16] Kim HK, Park SY, Lee JK, Oh TK (1998) Gene cloning and characterization of thermostable lipase from *Bacillus stearothermophilus* L1. Biosci Biotechnol Biochem 62: 66–71. 9501519 10.1271/bbb.62.66

[17] Li H, Zhang X (2005) Characterization of thermostable lipase from thermophilic *Geobacillus* sp. TW1. Protein Expr Purif 42: 153–159. 15939301 10.1016/j.pep.2005.03.011

[18] Leow TC, Rahman RN, Basri M, Salleh AB (2007) A thermoalkaliphilic lipase of *Geobacillus* sp. T1. Extremophiles : life under extreme conditions 11: 527–535. 17426920 10.1007/s00792-007-0069-y

[19] Dominguez A, Sanroman A, Fucinos P, Rua ML, Pastrana L, et al (2004) Quantification of intra- and extra-cellular thermophilic lipase/esterase production by *Thermus* sp. Biotechnol Lett 26: 705–708. 15195968 10.1023/b:bile.0000024092.27943.75

[20] Fucinos P, Dominguez A, Sanroman MA, Longo MA, Rua ML, et al (2005) Production of thermostable lipolytic activity by *Thermus* species. Biotechnol Prog 21: 1198–1205. 16080702 10.1021/bp050080g

[21] Lopez-Lopez O, Fucinos P, Pastrana L, Rua ML, Cerdan ME, et al (2010) Heterologous expression of an esterase from *Thermus thermophilus* HB27 in *Saccharomyces cerevisiae* . J Biotechnol 145: 226–232. 19961884 10.1016/j.jbiotec.2009.11.017

[22] Fucinos P, Atanes E, Lopez-Lopez O, Esperanza Cerdan M, Isabel Gonzalez-Siso M, et al (2011) Production and characterization of two N-terminal truncated esterases from *Thermus thermophilus* HB27 in a mesophilic yeast: effect of N-terminus in thermal activity and stability. Protein Expr Purif 78: 120–130. 21513802 10.1016/j.pep.2011.04.002

[23] du Plessis EM, Berger E, Stark T, Louw ME, Visser D (2010) Characterization of a novel thermostable esterase from *Thermus scotoductus* SA-01: evidence of a new family of lipolytic esterases. Curr Microbiol 60: 248–253. 19967376 10.1007/s00284-009-9533-5

[24] Salameh MA, Wiegel J (2007) Purification and characterization of two highly thermophilic alkaline lipases from *Thermosyntropha lipolytica* . Appl Environ Microbiol 73: 7725–7731. 17933930 10.1128/AEM.01509-07 PMC2168070

[25] Uppenberg J, Hansen MT, Patkar S, Jones TA (1994) The sequence, crystal structure determination and refinement of two crystal forms of lipase B from *Candida antarctica* . Structure 2: 293–308. 8087556 10.1016/s0969-2126(00)00031-9

[26] Gutarra ML, de Godoy MG, Silva Jdo N, Guedes IA, Lins U, et al (2009) Lipase production and *Penicillium simplicissimum* morphology in solid-state and submerged fermentations. Biotechnol J 4: 1450–1459. 19606429 10.1002/biot.200800298

[27] Bordes F, Tarquis L, Nicaud JM, Marty A (2011) Isolation of a thermostable variant of Lip2 lipase from *Yarrowia lipolytica* by directed evolution and deeper insight into the denaturation mechanisms involved. J Biotechnol 156: 117–124. 21763359 10.1016/j.jbiotec.2011.06.035

[28] Romdhane IB, Frikha F, Maalej-Achouri I, Gargouri A, Belghith H (2012) Gene cloning and molecular characterization of the *Talaromyces thermophilus* lipase catalyzed efficient hydrolysis and synthesis of esters. Gene 494: 112–118. 22178764 10.1016/j.gene.2011.11.059

[29] Siddiqui KS, Cavicchioli R (2005) Improved thermal stability and activity in the cold-adapted lipase B from *Candida antarctica* following chemical modification with oxidized polysaccharides. Extremophiles 9: 471–476. 15999221 10.1007/s00792-005-0464-1

[30] Tufvesson P, Annerling A, Hatti-Kaul R, Adlercreutz D (2007) Solvent-free enzymatic synthesis of fatty alkanolamides. Biotechnol Bioeng 97: 447–453. 17099913 10.1002/bit.21258

[31] Le QA, Joo JC, Yoo YJ, Kim YH (2012) Development of thermostable *Candida antarctica* lipase B through novel in-silico design of disulfide bridge. Biotechnol Bioeng. 10.1002/bit.24371 22095554

[32] Henne A, Schmitz RA, Bomeke M, Gottschalk G, Daniel R (2000) Screening of environmental DNA libraries for the presence of genes conferring lipolytic activity on *Escherichia coli* . Appl Environ Microbiol 66: 3113–3116. 10877816 10.1128/aem.66.7.3113-3116.2000 PMC92121

[33] Chow J, Krauss U, Jaeger K-E, Streit WR (2011) Carboxylesterases and Lipases from Metagenomes. In: Bruijn FJd, editor. Handbook of Molecular Microbial Ecology II : Metagenomics in Different Habitats. Hoboken, New Jersey: John Wiley & Sons Inc. 499–506.

[34] Schmeisser C, Steele H, Streit WR (2007) Metagenomics, biotechnology with non-culturable microbes. Appl Microbiol Biotechnol75: 955–962. 10.1007/s00253-007-0945-5 17396253

[35] Perner M, Ilmberger N, Köhler HU, Chow J, Streit WR (2011) Emerging Fields in Functional Metagenomics and Its Industrial Relevance: Overcoming Limitations and Redirecting the Search for Novel Biocatalysts. In: Bruijn FJd, editor. Handbook of Molecular Microbial Ecology II: John Wiley & Sons, Inc. 481–498.

[36] Handelsman J (2004) Metagenomics: application of genomics to uncultured microorganisms. Microbiol Mol Biol Rev 68: 669–685. 15590779 10.1128/MMBR.68.4.669-685.2004 PMC539003

[37] Liles MR, Williamson LL, Rodbumrer J, Torsvik V, Parsley LC, et al. (2009) Isolation and cloning of high-molecular-weight metagenomic DNA from soil microorganisms. Cold Spring Harbor protocols 2009: pdb prot5271. 10.1101/pdb.prot5271 20147247

[38] Reymond J-L (2006) Enzyme Assays: High-throughput Screening, Genetic Selection and Fingerprinting. Weinheim: WILEY-VCH Verlag GmbH & Co. KGaA.

[39] Nam KH, Kim SJ, Priyadarshi A, Kim HS, Hwang KY (2009) The crystal structure of an HSL-homolog EstE5 complex with PMSF reveals a unique configuration that inhibits the nucleophile Ser144 in catalytic triads. Biochem Biophys Res Commun 389: 247–250. 19715665 10.1016/j.bbrc.2009.08.123

[40] Nam KH, Kim MY, Kim SJ, Priyadarshi A, Kwon ST, et al (2009) Structural and functional analysis of a novel hormone-sensitive lipase from a metagenome library. Proteins 74: 1036–1040. 19089974 10.1002/prot.22313

[41] Fu J, Leiros HK, de Pascale D, Johnson KA, Blencke HM, et al. (2012) Functional and structural studies of a novel cold-adapted esterase from an Arctic intertidal metagenomic library. Appl Microbiol Biotechnol. 10.1007/s00253-012-4276-9 22832985

[42] Byun JS, Rhee JK, Kim ND, Yoon J, Kim DU, et al (2007) Crystal structure of hyperthermophilic esterase EstE1 and the relationship between its dimerization and thermostability properties. BMC Struct Biol 7: 47. 17625021 10.1186/1472-6807-7-47 PMC1936996

[43] Kim S, Joo S, Yoon HC, Ryu Y, Kim KK, et al (2007) Purification, crystallization and preliminary crystallographic analysis of Est25: a ketoprofen-specific hormone-sensitive lipase. Acta Crystallogr Sect F Struct Biol Cryst Commun 63: 579–581. 10.1107/S1744309107026152 PMC2335126 17620715

[44] Sako Y, Takai K, Ishida Y, Uchida A, Katayama Y (1996) *Rhodothermus obamensis* sp. nov., a modern lineage of extremely thermophilic marine bacteria. Int J Syst Bacteriol 46: 1099–1104. 8863442 10.1099/00207713-46-4-1099

[45] Castenholz RW (1969) Thermophilic blue-green algae and the thermal environment. Bacteriol Rev 33: 476–504. 4984428 10.1128/br.33.4.476-504.1969 PMC378340

[46] Brock TD, Freeze H (1969) *Thermus aquaticus* gen. n. and sp. n., a nonsporulating extreme thermophile. J Bacteriol 98: 289–297. 5781580 10.1128/jb.98.1.289-297.1969 PMC249935

[47] Sambrook J, Russel DW, editor (2001) Molecular cloning, a laboratory manual. New York, USA: Cold Spring Harbor Laboratory Press.

[48] Lawrence RC, Fryer TF, Reiter B (1967) Rapid method for the quantitative estimation of microbial lipases. Nature (London) 213: 1264–1265.

[49] Reetz MT, Zonta A, Schimossek K, Liebeton K, Jaeger KE (1997) Creation of enantioselective biocatalysts for organic chemistry by in vitro evolution. Angew Chem Int Ed Engl 36: 2830–2832.

[50] Franken BJ, Pietruszka J (2009) Protocols to screen for enantioselective lipases. In: Timmes KN, editor. Handbook of Microbiology of Hydrocarbons, Oils, Lipids, and Derived Compounds. Berlin-Heidelberg: Springer. 2860–2876.

[51] Pietruszka J, Simon RC, Kruska F, Braun M (2009) Dynamic Enzymatic Kinetic Resolution of Methyl 2,3-Dihydro-1H-indene-1-carboxylate. European J Org Chem: 6217–6224.

[52] Reetz MT, Prasad S, Carballeira JD, Gumulya Y, Bocola M (2010) Iterative Saturation Mutagenesis Accelerates Laboratory Evolution of Enzyme Stereoselectivity: Rigorous Comparison with Traditional Methods. J Am Chem Soc 132: 9144–9152. 20536132 10.1021/ja1030479

[53] Sandstrom AG, Wikmark Y, Engstrom K, Nyhlen J, Backvall JE (2012) Combinatorial reshaping of the *Candida antarctica* lipase A substrate pocket for enantioselectivity using an extremely condensed library. Proc Natl Acad Sci U S A 109: 78–83. 22178758 10.1073/pnas.1111537108 PMC3252943

[54] Salvadori P, Bertucci C, Rosini C (1991) Circular-Dichroism Detection in HPLC. Chirality 3: 376–385.

[55] Reetz MT, Kuhling KM, Hinrichs H, Deege A (2000) Circular dichroism as a detection method in the screening of enantioselective catalysts. Chirality 12: 479–482. 10824174 10.1002/(SICI)1520-636X(2000)12:5/6<479::AID-CHIR32>3.0.CO;2-#

[56] Hamzic M, Pietruszka J, Sandkuhl D (2011) HPLC-CD selectivity assay for alcohol dehydrogenases. Chirality 23 Suppl 1: E110–115. 21997932 10.1002/chir.21025

[57] Kroutil W, Kleewein A, Faber K (1997) A computer program for analysis, simulation and optimization of asymmetric catalytic processes proceeding through two consecutive steps. Type 2: sequential kinetic resolutions. Tetrahedron-Asymmetry 8: 3263–3274.

[58] Musidlowska A, Lange S, Bornscheuer UT (2001) By Overexpression in the Yeast *Pichia pastoris* to Enhanced Enantioselectivity: New Aspects in the Application of Pig Liver Esterase. Angew Chem Int Ed Engl 40: 2851–2853. 29712000 10.1002/1521-3773(20010803)40:15<2851::AID-ANIE2851>3.0.CO;2-V

[59] Musidlowska-Persson A, Bornscheuer UT (2002) Substrate specificity of the γ-isoenzyme of recombinant pig liver esterase towards acetates of secondary alcohols. J Mol Catal B Enzym 19–20: 129–133.

[60] Chen CS, Fujimoto Y, Girdaukas G, Sih CJ (1982) Quantitative-Analyses of Biochemical Kinetic Resolutions of Enantiomers. J Am Chem Soc 104: 7294–7299.

[61] Arpigny JL, Jaeger KE (1999) Bacterial lipolytic enzymes: classification and properties. Biochem J 343 Pt 1: 177–183. PMC1220539 10493927

[62] Notredame C, Higgins DG, Heringa J (2000) T-Coffee: A novel method for fast and accurate multiple sequence alignment. J Mol Biol 302: 205–217. 10964570 10.1006/jmbi.2000.4042

[63] Nicholas K, Nicholas HJ, Deerfield D (1997) GeneDoc: Analysis and Visualization of Genetic Variation. EMBNEWS 4: 14.

[64] Stamatakis A, Hoover P, Rougemont J (2008) A rapid bootstrap algorithm for the RAxML Web servers. Syst Biol 57: 758–771. 18853362 10.1080/10635150802429642

[65] Zmasek CM, Eddy SR (2001) ATV: display and manipulation of annotated phylogenetic trees. Bioinformatics 17: 383–384. 11301314 10.1093/bioinformatics/17.4.383

[66] Trooskens G, De Beule D, Decouttere F, Van Criekinge W (2005) Phylogenetic trees: visualizing, customizing and detecting incongruence. Bioinformatics 21: 3801–3802. 16030069 10.1093/bioinformatics/bti590

[67] Fersini F, Dall’antonia Y, Chow J, Streit WR, Mueller-Dieckmann J (2012) Cloning, expression, purification and preliminary X-ray analysis of a putative metagenome-derived lipase. Acta Crystallogr Sect F Struct Biol Cryst Commun 68: 923–926. 10.1107/S1744309112025651 PMC3412774 22869123

[68] Vagin A, Teplyakov A (1997) MOLREP: an automated program for molecular replacement. J Appl Crystallogr 30: 1022–1025.

[69] Matthews BW (1968) Solvent Content of Protein Crystals. J Mol Biol 33: 491–497. 5700707 10.1016/0022-2836(68)90205-2

[70] Emsley P, Lohkamp B, Scott WG, Cowtan K (2010) Features and development of Coot. Acta Crystallogr D Biol Crystallogr 66: 486–501. 20383002 10.1107/S0907444910007493 PMC2852313

[71] Murshudov GN, Vagin AA, Dodson EJ (1997) Refinement of macromolecular structures by the maximum-likelihood method. Acta Crystallogr D Biol Crystallogr 53: 240–255. 15299926 10.1107/S0907444996012255

[72] DeLano WL (2002) The PyMOL Molecular Graphics System. 1.5.0.1 ed. New York: Schroedinger LLC.

[73] Altschul SF, Madden TL, Schaffer AA, Zhang J, Zhang Z, et al (1997) Gapped BLAST and PSI-BLAST: a new generation of protein database search programs. Nucleic Acids Res 25: 3389–3402. 9254694 10.1093/nar/25.17.3389 PMC146917

[74] Nielsen H, Engelbrecht J, Brunak S, von Heijne G (1997) Identification of prokaryotic and eukaryotic signal peptides and prediction of their cleavage sites. Protein Eng 10: 1–6. 10.1093/protein/10.1.1 9051728

[75] Kazlauskas RJ (2006) Quantitative Assay of Hydrolases for Activity and Selectivity Using Color Changes. In: Reymond JL, editor. Enzyme-Assays: High-throughput Screening, Genetic Selection and Fingerprinting. Weinheim: WILEY-VCH Verlag GmbH & Co. KGaA. 16–39.

[76] Krissinel E, Henrick K (2007) Inference of macromolecular assemblies from crystalline state. J Mol Biol 372: 774–797. 17681537 10.1016/j.jmb.2007.05.022

[77] Ollis DL, Cheah E, Cygler M, Dijkstra B, Frolow F, et al (1992) The alpha/beta hydrolase fold. Protein Eng 5: 197–211. 1409539 10.1093/protein/5.3.197

[78] Holmquist M (2000) Alpha/Beta-hydrolase fold enzymes: structures, functions and mechanisms. Curr Protein Pept Sci 1: 209–235. 12369917 10.2174/1389203003381405

[79] Holm L, Rosenstrom P (2010) Dali server: conservation mapping in 3D. Nucleic Acids Res 38: W545–549. 20457744 10.1093/nar/gkq366 PMC2896194

[80] Liu P, Wang YF, Ewis HE, Abdelal AT, Lu CD, et al (2004) Covalent reaction intermediate revealed in crystal structure of the *Geobacillus stearothermophilus* carboxylesterase Est30. J Mol Biol 342: 551–561. 15327954 10.1016/j.jmb.2004.06.069

[81] Bennett MD, Delabre M-L, Holland R, Norris GE to be published.

[82] Goldstone DC, Villas-Boas SG, Till M, Kelly WJ, Attwood GT, et al (2010) Structural and functional characterization of a promiscuous feruloyl esterase (Est1E) from the rumen bacterium *Butyrivibrio proteoclasticus* . Proteins 78: 1457–1469. 20058325 10.1002/prot.22662

[83] Schalk-Hihi C, Schubert C, Alexander R, Bayoumy S, Clemente JC, et al (2011) Crystal structure of a soluble form of human monoglyceride lipase in complex with an inhibitor at 1.35 A resolution. Protein Sci 20: 670–683. 21308848 10.1002/pro.596 PMC3081545

[84] Lai KK, Stogios PJ, Vu C, Xu X, Cui H, et al (2011) An inserted alpha/beta subdomain shapes the catalytic pocket of *Lactobacillus johnsonii* cinnamoyl esterase. PLoS One 6: e23269. 21876742 10.1371/journal.pone.0023269 PMC3158066

[85] Labar G, Bauvois C, Borel F, Ferrer JL, Wouters J, et al (2010) Crystal structure of the human monoacylglycerol lipase, a key actor in endocannabinoid signaling. Chembiochem 11: 218–227. 19957260 10.1002/cbic.200900621

[86] Heikinheimo P, Goldman A, Jeffries C, Ollis DL (1999) Of barn owls and bankers: a lush variety of alpha/beta hydrolases. Structure 7: R141–146. 10404588 10.1016/s0969-2126(99)80079-3

[87] Dundas J, Ouyang Z, Tseng J, Binkowski A, Turpaz Y, et al (2006) CASTp: computed atlas of surface topography of proteins with structural and topographical mapping of functionally annotated residues. Nucleic Acids Res 34: W116–118. 16844972 10.1093/nar/gkl282 PMC1538779

[88] Voget S, Leggewie C, Uesbeck A, Raasch C, Jaeger KE, et al (2003) Prospecting for novel biocatalysts in a soil metagenome. Appl Environ Microbiol 69: 6235–6242. 14532085 10.1128/AEM.69.10.6235-6242.2003 PMC201203

[89] Daniel R (2005) The metagenomics of soil. Nat Rev Microbiol 3: 470–478. 15931165 10.1038/nrmicro1160

[90] Beloqui A, Nechitaylo TY, Lopez-Cortes N, Ghazi A, Guazzaroni ME, et al (2010) Diversity of glycosyl hydrolases from cellulose-depleting communities enriched from casts of two earthworm species. Appl Environ Microbiol 76: 5934–5946. 20622123 10.1128/AEM.00902-10 PMC2935051

[91] Schmidt O, Drake HL, Horn MA (2010) Hitherto unknown [Fe-Fe]-hydrogenase gene diversity in anaerobes and anoxic enrichments from a moderately acidic fen. Appl Environ Microbiol 76: 2027–2031. 20118375 10.1128/AEM.02895-09 PMC2838027

[92] Meilleur C, Hupe JF, Juteau P, Shareck F (2009) Isolation and characterization of a new alkali-thermostable lipase cloned from a metagenomic library. J Ind Microbiol Biotechnol 36: 853–861. 19333634 10.1007/s10295-009-0562-7

[93] Nishida H, Yun CS (2011) Phylogenetic and Guanine-Cytosine Content Analysis of *Symbiobacterium thermophilum* Genes. Int J Evol Biol 2011: 634505. 10.4061/2011/634505 PMC3039409 21350632

[94] Ohno M, Shiratori H, Park MJ, Saitoh Y, Kumon Y, et al (2000) *Symbiobacterium thermophilum* gen. nov., sp. nov., a symbiotic thermophile that depends on co-culture with a *Bacillus* strain for growth. Int J Syst Evol Microbiol 50: 1829–1832. 11034494 10.1099/00207713-50-5-1829

[95] Ueda K, Yamashita A, Ishikawa J, Shimada M, Watsuji T-o, et al (2004) Genome sequence of *Symbiobacterium thermophilum*, an uncultivable bacterium that depends on microbial commensalism. Nucleic Acids Res 32: 4937–4944. 15383646 10.1093/nar/gkh830 PMC519118

[96] Cava F, Hidalgo A, Berenguer J (2009) *Thermus thermophilus* as biological model. Extremophiles 13: 213–231. 19156357 10.1007/s00792-009-0226-6

[97] Balkwill DL, Kieft TL, Tsukuda T, Kostandarithes HM, Onstott TC, et al (2004) Identification of iron-reducing *Thermus* strains as *Thermus scotoductus* . Extremophiles 8: 37–44. 15064988 10.1007/s00792-003-0357-0

[98] Kieft TL, Fredrickson JK, Onstott TC, Gorby YA, Kostandarithes HM, et al (1999) Dissimilatory Reduction of Fe(III) and Other Electron Acceptors by a *Thermus* Isolate. Appl Environ Microbiol 65: 1214–1221. 10049886 10.1128/aem.65.3.1214-1221.1999 PMC91167

[99] Gounder K, Brzuszkiewicz E, Liesegang H, Wollherr A, Daniel R, et al (2011) Sequence of the hyperplastic genome of the naturally competent *Thermus scotoductus* SA-01. BMC Genomics 12: 577. 22115438 10.1186/1471-2164-12-577 PMC3235269

[100] Kristjansson JK, Hjorleifsdottir S, Marteinsson VT, Alfredsson GA (1994) *Thermus scotoductus*, sp. nov, a Pigment-Producing Thermophilic Bacterium from Hot Tap Water in Iceland and Including *Thermus* sp. X-1. Syst Appl Microbiol 17: 44–50.

[101] Skirnisdottir S, Hreggvidsson GO, Holst O, Kristjansson JK (2001) Isolation and characterization of a mixotrophic sulfur-oxidizing *Thermus scotoductus* . Extremophiles 5: 45–51. 11302502 10.1007/s007920000172

[102] Brock TD, Boylen KL (1973) Presence of thermophilic bacteria in laundry and domestic hot-water heaters. Appl Microbiol 25: 72–76. 4568892 10.1128/am.25.1.72-76.1973 PMC380738

[103] Kjeldsen KU, Kjellerup BV, Egli K, Frolund B, Nielsen PH, et al (2007) Phylogenetic and functional diversity of bacteria in biofilms from metal surfaces of an alkaline district heating system. FEMS Microbiol Ecol 61: 384–397. 17651138 10.1111/j.1574-6941.2006.00255.x

[104] Kjellerup BV, Thomsen TR, Nielsen JL, Olesen BH, Frolund B, et al (2005) Microbial diversity in biofilms from corroding heating systems. Biofouling 21: 19–29. 16019388 10.1080/08927010500070992

[105] Hutt AJ, Caldwell J (1984) The importance of stereochemistry in the clinical pharmacokinetics of the 2-arylpropionic acid non-steroidal anti-inflammatory drugs. Clin Pharmacokinet 9: 371–373. 6467769 10.2165/00003088-198409040-00007

[106] Gihani MT, Williams JM (1999) Dynamic kinetic resolution. Curr Opin Chem Biol 3: 11–15. 10021402 10.1016/s1367-5931(99)80003-9

[107] Kakugawa S, Fushinobu S, Wakagi T, Shoun H (2007) Characterization of a thermostable carboxylesterase from the hyperthermophilic bacterium *Thermotoga maritima* . Appl Microbiol Biotechnol74: 585–591. 10.1007/s00253-006-0687-9 17106678

