# Supplementary figures and images for: The Metagenome-Derived Enzymes LipS and LipT Increase the Diversity of Known Lipases
Source: PLoS One. 2012 Oct 24;7(10):e47665. doi: 10.1371/journal.pone.0047665 (PMC3480424; doi:10.1371/journal.pone.0047665)

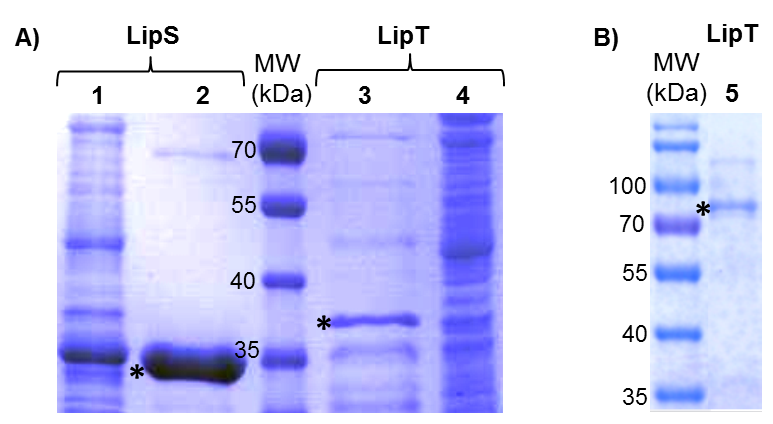

Supplement: Figure S1 — 15% SDS-PAGE of recombinant and purified LipS and LipT. Asterisks indicate the corresponding protein bands after His6-tag affinity chromatography. 15 µg of protein from the crude cell extracts or from the purified proteins were loaded and electrophoresed. A) 1, 4: crude cell extract; 2, 3 purified protein after extended heat treatment (30 min at 70°C and 5 min 95°C). B) Purified LipT after incomplete heat-denaturation (5 min 95°C). (TIF) [file pone.0047665.s001.tif]

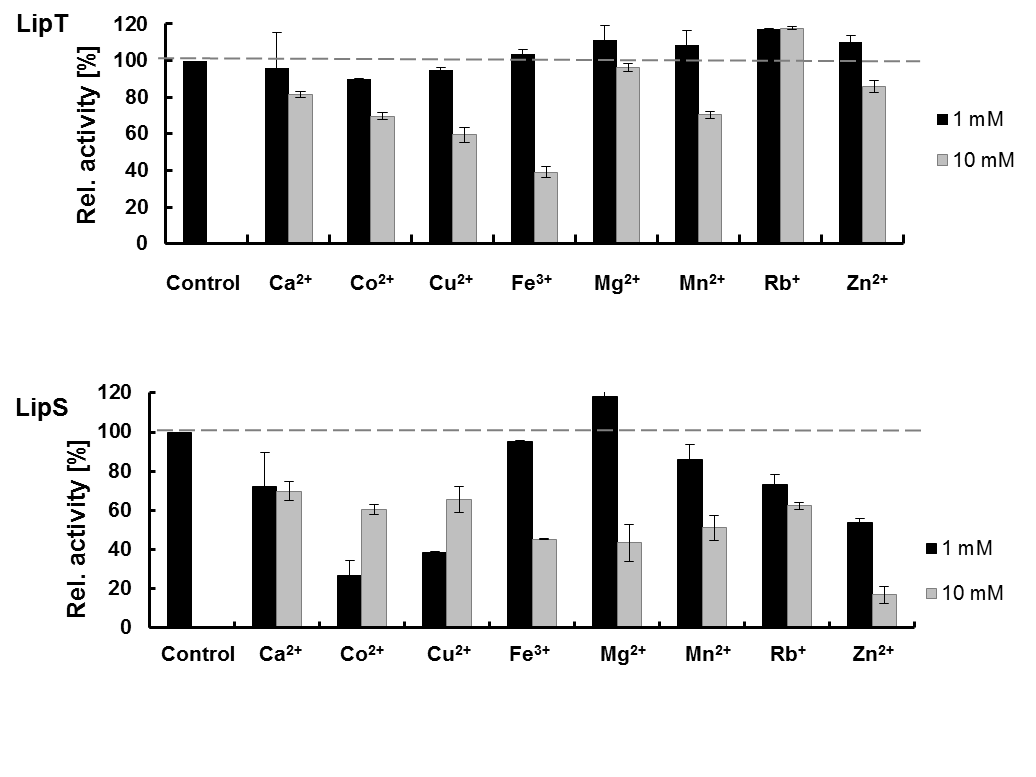

Supplement: Figure S2 — Effect of metal ions applied in 1 and 10 mM concentration on LipT and LipS. Residual activity of the enzymes was measured with pNP- dodecanoate at 75°C (LipT) and 70°C (LipS). Compared with the control without metal ions, none of the cations showed positive effects significant enough for being considered as cofactor. Data are mean values of at least three independent measurements and bars indicate the standard deviation. (TIF) [file pone.0047665.s002.tif]

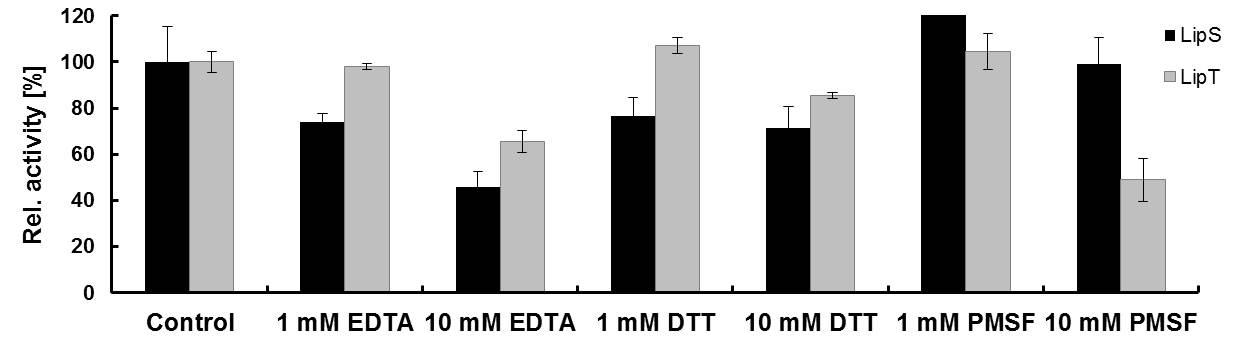

Supplement: Figure S3 — Effects of 1 and 10 mM EDTA, DTT and PMSF on the activity of LipS and LipT. The residual activity was measured at 70°C (LipS) and 75°C (LipT) using pNP-substrates. (TIF) [file pone.0047665.s003.tif]

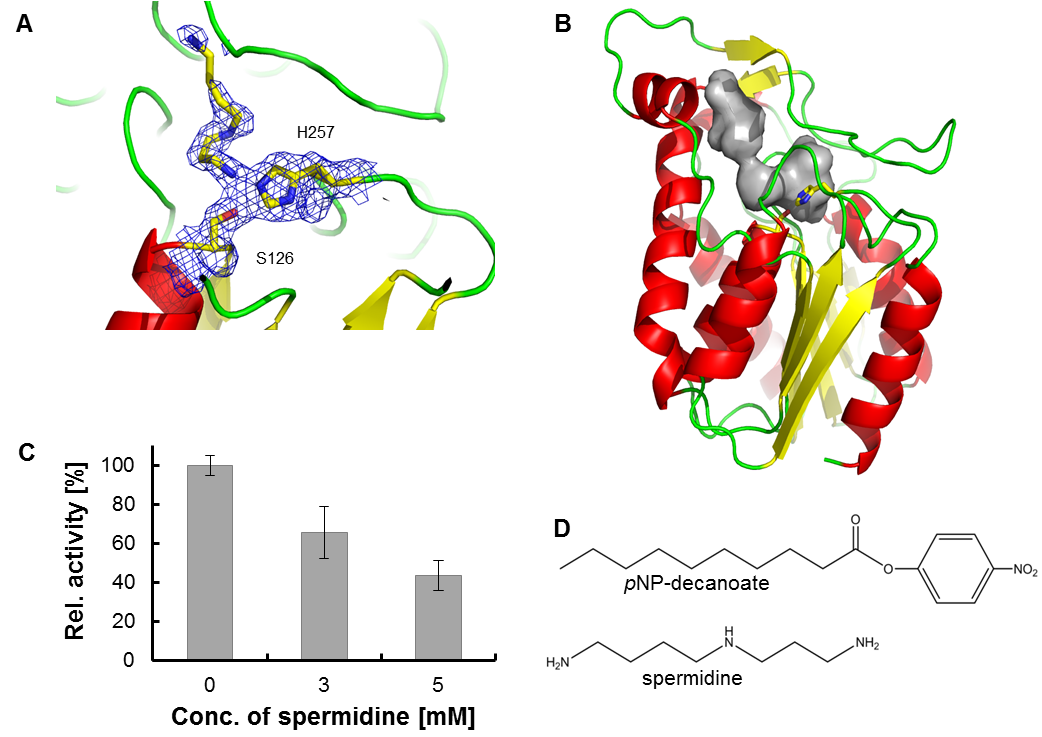

Supplement: Figure S4 — Effect of spermidine on the active site of LipS. A) Electron density maps (blue) around S126, H257 and spermidine. The additional density linking both residues and extending further towards the active site cavity was interpreted as spermidine. B) The spermidine moiety, shown as a space model colored in grey, is located in the active site cavity of LipS in vicinity of catalytic S126 and H257 indicated as sticks model. C) Spermidine inhibits LipS activity at concentrations of 3 and 5 mM compared to a control without added spermidine. Enzyme activity was determined after 5 min preincubation with spermidine using pNP-decanoate as substrate and incubation at 70°C for 10 min. D) Spermidine displays similarity with LipŚs substrate pNP-decanoate. (TIF) [file pone.0047665.s004.tif]

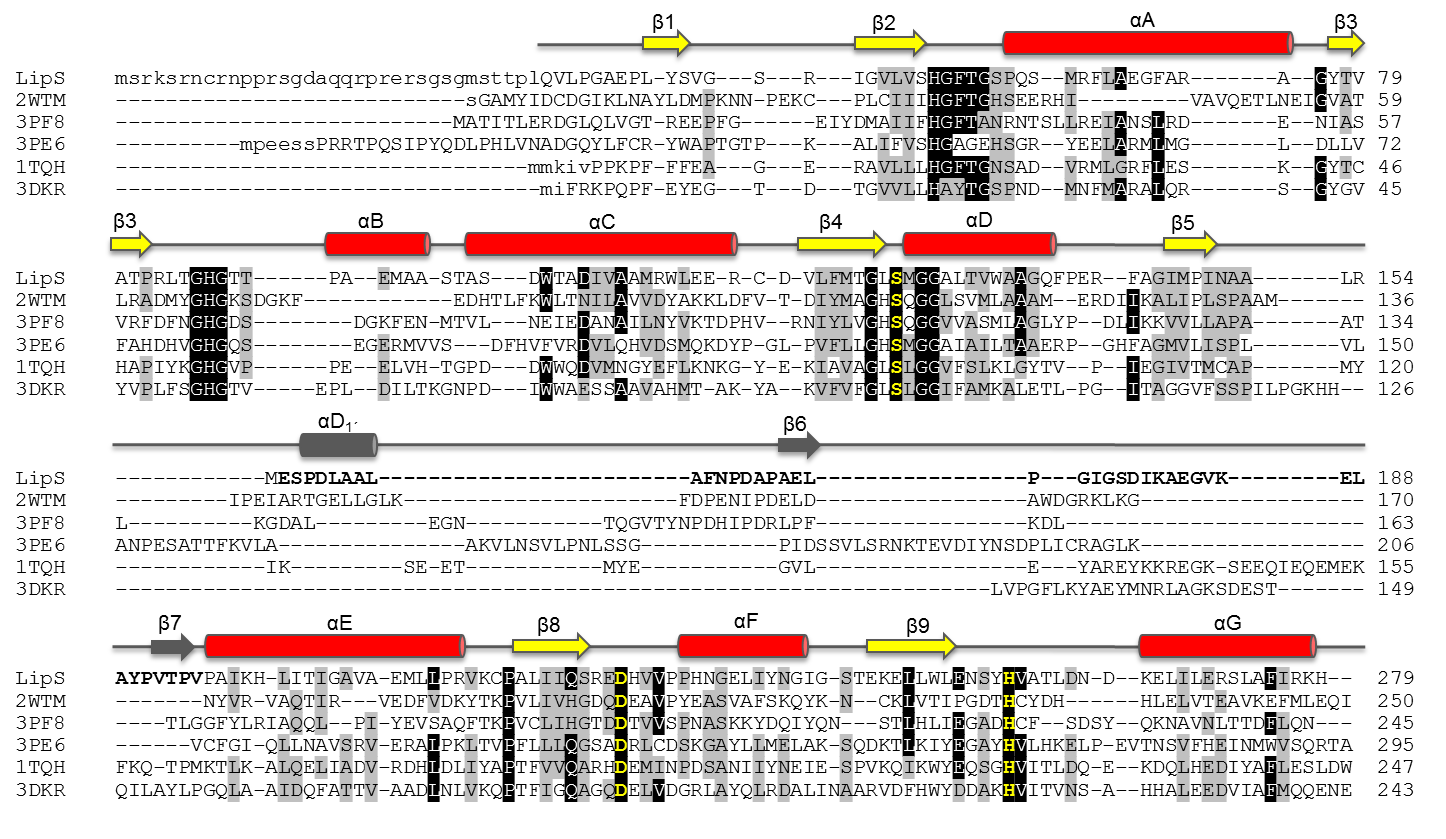

Supplement: Figure S5 — Structure based sequence alignment of LipS with its homologues. It revealed very low structural similarity in the region of the inserted domain, which is indicated by bold letters in LipS. 2WTM, Est1E form Butyrivibrio proteoclasticus [82]; 3PF8, LJ0536 from Lactobacillus johnsonii [84]; 3PE6, monoglyceride lipase (MGL) from Homo sapiens [83]; 1TQH, Est30 from Geobacillus stearothermophilus [80]; 3DKR, esterase D from Lactobacillus rhamnosus [81]. In the top line, secondary structure elements of LipS are shown with the inserted domain (αD1′, β6 and β7) colored in gray. Identical and similar amino acids conserved in at least four structures were shaded in black and gray respectively. Catalytic triad residues of LipS are indicated in bold and yellow. Residues which are not seen in the structures are shown as small letters. (TIF) [file pone.0047665.s005.tif]
