# Supplementary material for: The Metagenome-Derived Enzymes LipS and LipT Increase the Diversity of Known Lipases
Source: PLoS One. 2012 Oct 24;7(10):e47665. doi: 10.1371/journal.pone.0047665 (PMC3480424; doi:10.1371/journal.pone.0047665)
Supplement: Table S1 — Bacterial strains and plasmids used in this work. (DOCX) [file pone.0047665.s006.docx]

**SUPPORTING TABLE S1.** Bacterial strains and plasmids used in this work.

| Strain or construct | Description | Reference/source |
| --- | --- | --- |
|  |  |  |
| *E. coli* BL21 (DE3) | F^-^ *ompT hsdS*_B_ (r_B_^-^ m_B_^-^ ) *gal dcm* (DE3) | Novagen, Darmstadt, Germany |
| *E. coli* DH5α | *supE*44 Δ*lacU*169 (Ф80 lac*Z* ΔM15) *hsdR*17 *recA*1 *endA*1 *gyrA*96 *thi*-1 *relA*1 | Invitrogen, Karlsruhe, Germany |
| *E. coli* Epi100 | F^-^ *mcrA* Δ(*mrr-hsdRMS-mcrBC*) Φ80*dlacZ*ΔM15 Δ*lacX*74 *recA1* *endA1 araD139* Δ(*ara, leu*) 7697 *galU* *galK* λ-*rpsL nupG* | Epicentre (Madison, WI, USA) |
| pSuperCos | Cosmid-vector, amp^R^, neo^R^, *cos*, T3- and T7-promotor (7.9 kb) | Stratagene, La Jolla, CA, USA |
| pTZ19R::Cm^R^ | Cloning vector, *lacZ*, cm^R^, T7-promotor (3.14 kb) | Fermentas, St. Leon-Rot, Germany |
| pDrive | TA-cloning vector, *oriEc*, P_lac_*lacZ*, amp^R^, kan^R^, T7-promotor (3.85 kb) | Qiagen, Hilden, Germany |
| pET21a | Expression vector, *lacI*, amp^R^, T7-promotor, C-terminal His_6_-tag coding sequence (5.44 kb) | Novagen, Darmstadt, Germany |
| pETM11 | Crystallization vector, N- and C-terminal His_6_-tag coding sequences, TEV site (6.03 kb) | EMBL c/o DESY (Hamburg, Germany) |
| pCos6B1 | Cosmid clone with 27 kb insert in pSuperCos | This work |
| pCos9D12 | Cosmid clone with 26.5 kb insert in pSuperCos | This work |
| *lipT*::pET21a | *lipT* (0.99 kb), derived from pCos6B1, cloned into pET21a at *Nde*I and *Hind*III restriction sites | This work |
| *lipS*::pET21a | *lipS* (0.84 kb), derived from pCos9D12, cloned into pET21a at *Nde*I and *Hind*III restriction sites | This work |
| *lipS*:: pETM11 | *lipS* cloned into pETM11 with *Nco*I and *Hind*III restriction sites | This work |
