# Supplementary material for: The Metagenome-Derived Enzymes LipS and LipT Increase the Diversity of Known Lipases
Source: PLoS One. 2012 Oct 24;7(10):e47665. doi: 10.1371/journal.pone.0047665 (PMC3480424; doi:10.1371/journal.pone.0047665)
Supplement: Table S2 — Amino acid sequences of members from the eight major lipase and esterase families [61] received from GenBank together with members of the new LipS and LipT groups. (DOCX) [file pone.0047665.s007.docx]

**SUPPORTING TABLE S2.** Amino acid sequences of members from the eight major lipase and esterase families [61] received from GenBank together with members of the new LipS and LipT groups.

| Family | Sub-family | Organism | Annotation | GenBank acc. no. |
| --- | --- | --- | --- | --- |
| I | 1 | *Acinetobacter calcoaceticus* | Lipase | CAA56780.1 |
|  |  | *Pseudomonas aeruginosa* | Lipase | BAA09135.1 |
|  |  | *Pseudomonas fluorescens* | Lipase | AAC15585.1 |
|  |  | *Pseudomonas fragi* | Unnamed protein product | CAA32193.1 |
| I | 2 | *Burkholderia cepacia* | Lipase | AAA50466.1 |
|  |  | *Burkholderia glumae* | Lipase | CAA49812.1 |
|  |  | *Pseudomonas luteola* | Triacylglycerol lipase | O68551 |
| I | 3 | *Pseudomonas fluorescens* | Triacylglycerol lipase | BAA02012.1 |
|  |  | *Serratia marcescens* | Lipase | BAA02519.1 |
| I | 4 | *Bacillus megaterium* | Extracellular esterase precursor | Q8RJP5 |
|  |  | *Bacillus pumilus* | Lipase | CAA02196.1 |
|  |  | *Bacillus subtilis* | Lipase | AAA22574.1 |
| I | 5 | *Geobacillus stearothermophilus* | Lipase | AAC12257.1 |
|  |  | *Geobacillus thermocatenulatus* | Triacylglycerol lipase | CAA64621.1 |
|  |  | *Geobacillus zalihae* | Thermostable lipase | Q842J9 |
| I | 6 | *Staphylococcus haemolyticus* | Lipase | Q9RGZ6 |
|  |  | *Staphylococcus hyicus* | Triacylglycerol lipase | P04635.1 |
| I | 7 | *Corynebacterium glutamicum* | Triacylglycerol lipase | Q8NU60 |
|  |  | *Propionibacterium acnes* | Triacylglycerol lipase | CAA67627.1 |
|  |  | *Streptomyces cinnamoneus* | Lipase | AAB71210.1 |
| I | 8 | *Colwellia psychrerythraea* | Hypothetical protein | Q48AN1 |
|  |  | *Hahella chejuensis* | Putative esterase/lipase | Q2SGZ8 |
|  |  | *Pseudoalteromonas haloplanktis* | Hypothetical protein | Q3IF07 |
| II (GDSL) |  | *Aeromonas hydrophila* | Phosphatidylcholine-sterol acyltransferase | P10480.3 |
|  |  | *Xanthomonas vesicatoria* | Esterase EstE | Q7X4K7 |
|  |  | *Photorhabdus luminescens* | Triacylglycerol lipase | CAA47020.1 |
|  |  | *Pseudomonas aeruginosa* PAO1 | Lipase/esterase | AAB61674.1 |
|  |  | *Salmonella enterica* subsp. *enterica* serovar *typhimurium* | Outer membrane esterase | AAC38796.1 |
| III |  | *Moraxella* sp. | Unnamed protein product | CAA37220.1 |
|  |  | *Streptomyces* sp. | Triacylglycerol hydrolase | AAB51445.1 |
| IV (HSL) |  | *Streptomyces* sp. | Hydrolase | Q7SIG1 |
|  |  | *Cupriavidus necator* | Lipase-like enzyme | AAC41424.1 |
|  |  | *Moraxella* sp. | Triacylglycerol lipase | CAA37862.1 |
|  |  | *Pseudomonas* sp. B11-1 | Lipase | AAC38151.1 |
| V |  | *Pseudomonas oleovorans* | PHA-depolymerase | AAA25933.1 |
|  |  | *Psychrobacter immobilis* | Triacylglycerol lipase | CAA47949.1 |
|  |  | *Sulfolobus acidocaldarius* | Lipolytic enzyme | AAC67392.1 |
| VI |  | *Arthrospira platensis* | Serine esterase | AAB30793.1 |
|  |  | *Pseudomonas fluorescens* | Esterase II | AAC60403.1 |
|  |  | *Rickettsia prowazekii* | Esterase | CAA72452.1 |
|  |  | *Xanthomonas campestris* | Q3BXV6_XANC5 | Q3BXV6 |
| VII |  | *Arthrobacter oxydans* | Phenmedipham hydrolase | Q01470.1 |
|  |  | *Bacillus subtilis* | *para*-nitrobenzyl esterase | P37967.2 |
|  |  | *Streptomyces coelicolor* A3(2) | Putative carboxylesterase | CAA22794.1 |
| VIII |  | *Arthrobacter globiformis* | Carboxylic ester hydrolase | AAA99492.1 |
|  |  | *Pseudomonas fluorescens* | Esterase III | AAC60471.2 |
|  |  | *Streptomyces anulatus* | Esterase A | CAA78842.1 |
| LipS Group |  | *Rhodopseudomonas palustris* DX-1 | Acylglycerol lipase | ADU46368.1 |
|  |  | *Symbiobacterium thermophilum* IAM 14863 | Esterase | BAD41030.1 |
|  |  | *Bacillus* sp. 2_A_57_CT2 | Esterase | EFV74766.1 |
|  |  | *Bacillus* sp. NRRL B-14911 | Esterase | EAR67363.1 |
|  |  | *Geobacillus thermodenitrificans* | EstGtA2 esterase | AEN92268.1 |
|  |  | *Rhodopseudomonas palustris* CGA009 | Putative carboxylesterase | CAE30086.1 |
| LipT Group |  | *Thermus aquaticus* Y51MC23 | Putative esterase | EED09760.1 |
|  |  | *Thermus scotoductus* SA-01 | Putative esterase | ADW21422.1 |
|  |  | *Thermus* sp. CCB_US3_UF1 | Hypothetical protein TCCBUS3UF1_8960 | AEV15941.1 |
|  |  | *Thermus thermophilus* HB27 | Putative esterase | AAS81248.1 |
