# Supplementary material for: The Metagenome-Derived Enzymes LipS and LipT Increase the Diversity of Known Lipases
Source: PLoS One. 2012 Oct 24;7(10):e47665. doi: 10.1371/journal.pone.0047665 (PMC3480424; doi:10.1371/journal.pone.0047665)
Supplement: Table S3 — Metagenomic esterases and lipases from uncultured organisms grouped into existing families [61] . The classification is based on alignment scores with members of family I-VIII or unknown families (UF). UF1-5: Unknown Families of metagenomic esterases/lipases: Sequences were grouped together into one family if at least two unclassified sequences shared significant similarity and sufficiently high T-COFFEE alignment scores. All sequences considered here as unclassified cannot be unequivocally grouped into any of the known other eight lipase/esterase families. *:I/S/G: Identical/Similar/Gapped amino acid positions in the respective alignment. Sequence identity/similarity is given with respect to one of the sequences of a known organism. Identity/similarity can be higher to metagenomic sequences in the subfamily. (DOCX) [file pone.0047665.s008.docx]

**SUPPORTING TABLE S3.** Metagenomic esterases and lipases from uncultured organisms grouped into existing families [61]. The classification is based on alignment scores with members of family I-VIII or unknown families (UF).

| Fam. | Tree-ID | Annotation | I/S/G* | to Organism (acc. no.) | Annota-tion |
| --- | --- | --- | --- | --- | --- |
| I | LipC12 (AEK97793.1) | Lipase | 52/69/6 | *Pseudomonas fragii* (CAA32193.1) | Unknown |
|  | EstAS (ACJ13070.1) | Lipase | 20/31/27 | *Vibrio harveyi* HY01 (EDL68452.1) | Lipase |
|  | LipB (AAP76489.1) | Lipase | 61/76/1 | *Serratia marcescens* (BAA02519.1) | Lipase |
|  | LipA (JQ360624) | Lipase (*Geobacillus* sp. HH01) | 97/98/0 | *Geobacillus stearothermophilus* (AAC12257.1) | Lipase |
|  | Est19 (AEM45127.1) | Hypothetical lipase | 29/42/12 | *Propionibacterium acnes* (CAA67627.1) | Triacylgly-cerol lipase |
| III | LipIAF5.2 (ACC95208.1) | Lipase | 41/54711 | *Streptomyces* sp. (AAB51445.1) | Triacylgly-cerol hydrolase |
| IV | Orf112 (AAF87664.1) | Esterase | 23/36/12 | *Pseudomonas* sp. B11-1 (AAC38151.1) | Lipase |
|  | EstPS2 (ACU30828.1) | Esterase | 24/38/9 | *Alicyclobacillus acidocaldarius* (Q7SIG1) | Hydrolase |
|  | EstKT4 (ABY61092.1) | Esterase | 24/41/7 | *Alicyclobacillus acidocaldarius* (Q7SIG1) | Hydrolase |
|  | EstKT7 (ADH59413.1) | Esterase | 18/33/18 | *Alicyclobacillus acidocaldarius* (Q7SIG1) | Hydrolase |
|  | EstKT9 (ADH59414.1) | Esterase | 17/31/23 | *Alicyclobacillus acidocaldarius* (Q7SIG1) | Hydrolase |
|  | EstB (ABY60417.1) | Esterase | 22/38/13 | *Alicyclobacillus acidocaldarius* (Q7SIG1) | Hydrolase |
|  | h1Lip1 (AAZ67909.1) | Lipase | 21/35/15 | *Alicyclobacillus acidocaldarius* (Q7SIG1) | Hydrolase |
|  | EstMY (ADM67446.1) | Esterase | 26/41/12 | *Alicyclobacillus acidocaldarius* (Q7SIG1) | Hydrolase |
|  | Est30 (AEM45138.1) | Hypothetical protein | 19/29/22 | *Alicyclobacillus acidocaldarius* (Q7SIG1) | Hydrolase |
| V | RlipE2 (ACM91115.1) | Lipase | 15/26/33 | *Psychrobacter immobilis* (CAA47949.1) | Triacylgly-cerol lipase |
|  | Est13 (AEM45121.1) | Hypothetical protein | 15/29/24 | *Psychrobacter immobilis* (CAA47949.1) | Triacylgly-cerol lipase |
|  | PWTSB (ABY52801.1) | Putative lipase | 56/63/15 | *Psychrobacter immobilis* (CAA47949.1) | Triacylgly-cerol lipase |
| VI | Est 21 (AEM45129.1) | Hypothetical protein | 48/65/3 | *Xanthomonas campestris* pv. *vesicatoria* (strain 85-10, Q3BXV6) | Carboxyl-esterase |
| VIII | EstA3 (AAZ48934.1) | Putative β-lactamase class C (WWRS) | 16/30/22 | *Arthrobacter globiformis* (AAA99492.1) | Carboxylic ester hydrolase |
|  | EstM N2 (AEA07655.1) | Esterase | 16/30/19 | *Streptomyces anulatus* (CAA78842.1) | Esterase A |
|  | EstCE1 (AAY90130.1) | Esterase (pCosCE1) | 19/31/15 | *Streptomyces anulatus* (CAA78842.1) | Esterase A |
|  | Est2K (ACX51146.1) | Est2K precursor | 15/29/23 | *Streptomyces anulatus* (CAA78842.1) | Esterase A |
|  | Est08 (AEM45116.1) | Hypothetical protein | 33/48/4 | *Streptomyces anulatus* (CAA78842.1) | Esterase A |
|  | LR1 (AAZ32715.1) | Lipase/ esterase | 19/32/18 | *Streptomyces anulatus* (CAA78842.1) | Esterase A |
|  | EstC (ACH88047.1) | EstC | 18/30/23 | *Streptomyces anulatus* (CAA78842.1) | Esterase A |
|  | EstM N1 (AEA07653.1) | EstC | 16/29/15 | *Streptomyces anulatus* (CAA78842.1) | Esterase A |

| Unknown | Tree-ID | Annotation | Fam. | I/S/G |
| --- | --- | --- | --- | --- |
| UF1 | Orf111 (AAF87663.1) | Esterase | UF1 | 25/37/33 |
|  | EstY (ABY83635.1) | Esterase | UF1 | 25/37/33 |
| UF2 | EstGK1 (ADE28719.1) | Esterase | UF3 | 61/75/1 |
|  | EstZ3 (ADE28720.1) | Esterase | UF3 | 61/75/1 |
|  | EstD2 (ADN26553.1) | Esterase | UF3 | 15/28/36 |
| UF3 | CHA3 (ACF94292.1) | Esterase | UF4 | 21/31/35 |
|  | RlipE1 (ACM91105.1) | Lipase (Rlip1) | UF4 | 21/31/35 |
| UF4 | Orf113 (AAF87665.1) | Esterase | UF5 | 18/27/28 |
|  | Est15 (AEM45123.1) | Hypothetical protein | UF5 | 18/27/28 |
| UF5 | LipA (AAF87662.1) | Lipase | UF6 | 15/27/29 |
|  | LipG (ABE69172.1) | Probable lipase (pFosLip) | UF6 | 15/27/29 |

**UF1-5:** Unknown Families of metagenomic esterases/lipases: Sequences were grouped together into one family if at least two unclassified sequences shared significant similarity and sufficiently high T-COFFEE alignment scores.

All sequences considered here as unclassified cannot be unequivocally grouped into any of the known other eight lipase/esterase families.

***:I/S/G:** Identical/Similar/Gapped amino acid positions in the respective alignment. Sequence identity/similarity is given with respect to one of the sequences of a known organism. Identity/similarity can be higher to metagenomic sequences in the subfamily.
