# Supplementary material for: The Metagenome-Derived Enzymes LipS and LipT Increase the Diversity of Known Lipases
Source: PLoS One. 2012 Oct 24;7(10):e47665. doi: 10.1371/journal.pone.0047665 (PMC3480424; doi:10.1371/journal.pone.0047665)
Supplement: Table S4 — Refinement and quality statistics of the crystallized constructs LipS-H6 and LipS-WT. (DOCX) [file pone.0047665.s009.docx]

**SUPPORTING TABLE S4.** Refinement and quality statistics of the crystallized constructs LipS-H6 and LipS-WT.

|  | LipS-H6 | LipS-WT |
| --- | --- | --- |
| Resolution (Å) | 2.80 | 1.99 |
| No. reflections | 16386 | 90469 |
| *R*_work_ / *R*_free_ (%) | 22.26 / 27.02 | 17.60 / 21.68 |
| No. atoms |  |  |
| Protein | 3779 | 7449 |
| Ligand/ion | 2 | 34 |
| Water | 37 | 608 |
| *B*-factors |  |  |
| Protein | 24.93 | 27.99 |
| Ligand/ion | 38.95 | 40.26 |
| Water | 10.50 | 36.60 |
| R. m. s. deviations |  |  |
| Bond lengths (Å) | 0.016 | 0.029 |
| Bond angles (°) | 1.649 | 2.193 |
