# Supplementary material for: The Metagenome-Derived Enzymes LipS and LipT Increase the Diversity of Known Lipases
Source: PLoS One. 2012 Oct 24;7(10):e47665. doi: 10.1371/journal.pone.0047665 (PMC3480424; doi:10.1371/journal.pone.0047665)
Supplement: Table S5 — Purification table of the proteins LipS and LipT. (1) Crude cell extract; (2) heat denaturation at 70°C for 30 min; (3) immobilized metal ion affinity chromatography with Ni-ions. In case of LipT, the elution fractions have been combined and concentrated to a residual volume of 0.4 ml in a centrifuge (Vivaspin20, MWCO 10,000; Sartorius Stedim Biotech GmbH, Göttingen, Germany; Centrifuge 5804R, rotor A-4-44, Eppendorf, Hamburg, Germany). Activity was measured using 0.5 mM pNP-octanoate at 70°C (LipS) or 0.5 mM pNP-decanoate at 75°C (LipT). (DOCX) [file pone.0047665.s010.docx]

**SUPPORTING TABLE S5.** Purification table of the proteins LipS and LipT.

| LipS |  |  |  |  |  |  |
| --- | --- | --- | --- | --- | --- | --- |
| Step | **Volume**  **[ml]** | **Total activity**  **[U]** | **Total protein**  **[mg]** | **Specific activity**  **[U/mg]** | **Yield**  **[%]** | **Purification**  **factor** |
| CE (1) | 4.0 | 36.3 | 47.7 | 0.8 | 100 | -- |
| HD (2) | 3.8 | 7.7 | 8.2 | 0.9 | 21 | 1.2 |
| IMAC (3) | 2.0 | 27.8 | 2.0 | 13.7 | 77 | 18.1 |

| LipT |  |  |  |  |  |  |
| --- | --- | --- | --- | --- | --- | --- |
| Step | **Volume**  **[ml]** | **Total activity**  **[U]** | **Total protein**  **[mg]** | **Specific activity**  **[U/mg]** | **Yield**  **[%]** | **Purification**  **factor** |
| CE (1) | 4.0 | 5.14 | 36.7 | 0.14 | 100 | -- |
| HD (2) | 3.8 | 0.04 | 4.3 | 0.01 | 1 | 0.1 |
| IMAC (3) | 0.4 | 0.18 | 1.8 | 0.10 | 4 | 0.7 |

(1) Crude cell extract; (2) heat denaturation at 70°C for 30 min; (3) immobilized metal ion affinity chromatography with Ni-ions. In case of LipT, the elution fractions have been combined and concentrated to a residual volume of 0.4 ml in a centrifuge (Vivaspin20, MWCO 10,000; Sartorius Stedim Biotech GmbH, Göttingen, Germany; Centrifuge 5804R, rotor A-4-44, Eppendorf, Hamburg, Germany). Activity was measured using 0.5 mM *p*NP-octanoate at 70 °C (LipS) or 0.5 mM *p*NP-decanoate at 75 °C (LipT).
