# Supplementary material for: The Metagenome-Derived Enzymes LipS and LipT Increase the Diversity of Known Lipases
Source: PLoS One. 2012 Oct 24;7(10):e47665. doi: 10.1371/journal.pone.0047665 (PMC3480424; doi:10.1371/journal.pone.0047665)
Supplement: Table S6 — Residual activities of LipS and LipT in the presence of organic solvents. The enzymes were incubated for 1 h at room temperature with the solvents diluted in 0.1 M PB pH 8.0 before pNP-dodecanoate was added as substrate. After incubation for 10 min at 70°C (LipS) and 75°C (LipT), the reaction was measured in a photometer at 405 nm against an enzyme-free blank containing the respective solvent and concentration. Data are mean values of at least three independent measurements; ± indicates the standard deviation. (DOCX) [file pone.0047665.s011.docx]

**SUPPORTING TABLE S6.** Residual activities of LipS and LipT in the presence of organic solvents. The enzymes were incubated for 1 h at room temperature with the solvents diluted in 0.1 M PB pH 8.0 before *p*NP-dodecanoate was added as substrate. After incubation for 10 min at 70 °C (LipS) and 75 °C (LipT), the reaction was measured in a photometer at 405 nm against an enzyme-free blank containing the respective solvent and concentration.

| Solvent added (vol/vol) | Relative Activities in % | |
| --- | --- | --- |
|  | LipS LipT | |
| none | 100 ± 5.45 | 100 ± 4.88 |
| DMSO 10% | 65.45 ± 3.64 | 156.52 ± 1.70 |
| 30% | 92.93 ± 8.48 | 100.11 ± 1.48 |
| Isopropanol 10% | 64.85 ± 2.63 | 49.21 ± 4.88 |
| 30% | 45.86 ± 2.63 | 6.15 ± 1.48 |
| Methanol 10% | 67.88 ± 2.02 | 108.46 ± 3.28 |
| 30% | 31.31 ± 0.61 | 57.47 ± 5.03 |
| DMF 10% | 31.92 ± 9.70 | 98.78 ± 5.79 |
| 30% | 16.97 ± 9.29 | 47.71 ± 2.90 |
| Acetone 10% | 39.80 ± 12.73 | 102.97 ± 14.10 |
| 30% | 8.48 ± 0.40 | 50.69 ± 1.06 |
| Acetonitrile 10% | 67.27 ± 5.86 | 96.29 ± 3.61 |
| 30% | 15.35 ± 0.40 | 50.90 ± 2.12 |
| Ethanol 10% | 27.68 ± 5.86 | 87.81 ± 15.27 |
| 30% | 8.89 ± 8.08 | 57.79 ± 3.08 |

Data are mean values of at least three independent measurements; ± indicates the standard deviation.
