# Supplementary material for: The Metagenome-Derived Enzymes LipS and LipT Increase the Diversity of Known Lipases
Source: PLoS One. 2012 Oct 24;7(10):e47665. doi: 10.1371/journal.pone.0047665 (PMC3480424; doi:10.1371/journal.pone.0047665)
Supplement: Table S7 — Amino acids and atoms building the active site pocket of LipS. Amino acids belonging to the inserted domain are indicated in bold. (DOCX) [file pone.0047665.s012.docx]

**SUPPORTING TABLE S7.** Amino acids and atoms building the active site pocket of LipS. Amino acids belonging to the inserted domain are indicated in bold.

| Amino acid | Atom |
| --- | --- |
| GLY57 | C, Cα, N |
| PHE58 | Cβ, Cε2, N, O |
| THR59 | Oγ1 |
| GLY60 | Cα, N |
| SER64 | Oγ |
| LEU125 | Cδ2 |
| SER126 | Cβ, Oγ |
| MET127 | Cβ, N |
| ALA152 | Cβ |
| MET155 | Cβ, Cε, O, Sδ |
| SER157 | **Cβ, Oγ** |
| ASP159 | **Cβ, Oδ2** |
| LEU160 | **Cα, Cβ, Cδ1, Cδ2, N** |
| LEU163 | **Cδ1** |
| LEU173 | **Cβ, Cδ1, Cδ2** |
| PRO174 | **Cβ, Cγ, O** |
| GLY175 | **C, O** |
| ILE176 | **Cα, Cβ, Cδ1, Cγ1, Cγ2, O** |
| GLY177 | **Cα** |
| ASP179 | **Cβ, N** |
| GLU187 | **Oε1, Oε2** |
| ALA189 | **Cβ** |
| THR193 | **Cβ, Cγ2, Oγ1** |
| ILE198 | Cδ1, Cγ1 |
| LEU201 | Cδ1, Cδ2 |
| HIS228 | O |
| VAL229 | Cα, Cγ1, Cγ2, O |
| VAL230 | Cγ2 |
| HIS257 | Cδ2, Nε2 |
| VAL258 | Cγ2 |
